# Physical performance testing in climbing—A systematic review

**DOI:** 10.3389/fspor.2023.1130812

**Published:** 2023-05-09

**Authors:** Kaja Langer, Christian Simon, Josef Wiemeyer

**Affiliations:** Laboratory for Movement & Exercise Science, Institute of Sports Science, Department of Human Sciences, Technical University of Darmstadt, Darmstadt, Germany

**Keywords:** performance, strength, endurance, flexibility, bouldering, testing, measuring

## Abstract

Due to the increasing popularity of climbing, the corresponding diagnostics are gaining in importance for both science and practice. This review aims to give an overview of the quality of different diagnostic testing- and measurement methods for performance, strength, endurance, and flexibility in climbing. A systematic literature search for studies including quantitative methods and tests for measuring different forms of strength, endurance, flexibility, or performance in climbing and bouldering was conducted on PubMed and SPORT Discus. Studies and abstracts were included if they a) worked with a representative sample of human boulderers and/or climbers, b) included detailed information on at least one test, and c) were randomized-controlled-, cohort-, cross-over-, intervention-, or case studies. 156 studies were included into the review. Data regarding subject characteristics, as well as the implementation and quality of all relevant tests were extracted from the studies. Tests with similar exercises were grouped and the information on a) measured value, b) unit, c) subject characteristics (sex and ability level), and d) quality criteria (objectivity, reliability, validity) were bundled and displayed in standardized tables. In total, 63 different tests were identified, of which some comprised different ways of implementation. This clearly shows that there are no uniform or standard procedures in climbing diagnostics, for tests on strength, endurance or flexibility. Furthermore, only few studies report data on test quality and detailed information on sample characteristics. This not only makes it difficult to compare test results, but at the same time makes it impossible to give precise test recommendations. Nevertheless, this overview of the current state of research contributes to the creation of more uniform test batteries in the future.

## 1. Introduction

**Figure 1:**
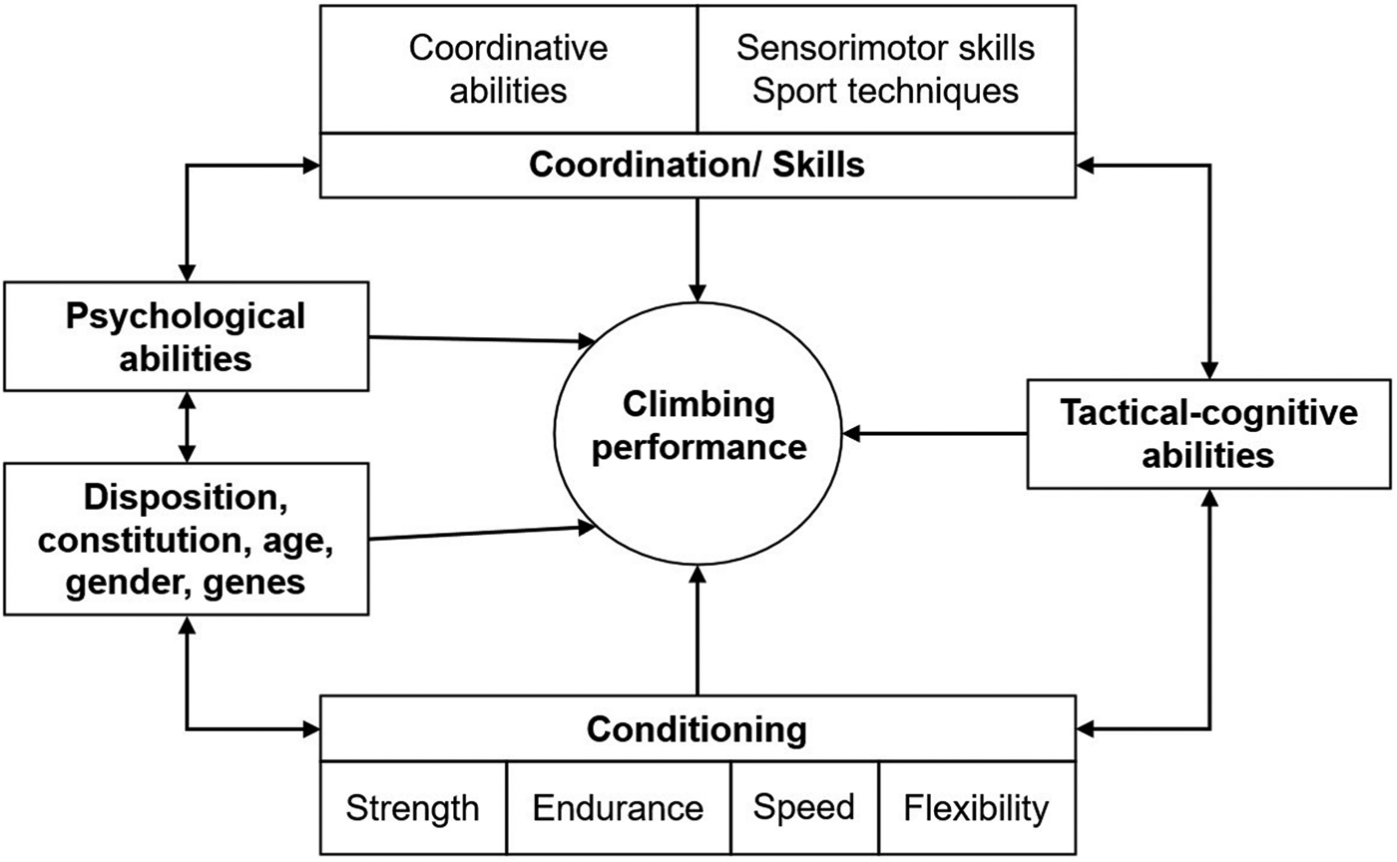
Performance structure of climbing (own figure).

Climbing (lead climbing, speed climbing, bouldering) has become an increasingly popular sport attracting a growing number of researchers around the world. This has led to a constantly growing database with many insights into the performance-determining factors of climbing. A broad overview of this is given in Figure 1.

It has been shown that performance in climbing and bouldering depends on psychological, skill-related, anthropometric, tactical-cognitive, and on conditional factors (1). As shown by MacLeod et al. (2), Grant et al. (3), Laffaye et al. (4), and Saul et al. (1), one of the most important conditional factors in climbing is finger strength. Moreover, MacLeod et al. (2) found greater finger endurance in intermittent tests in climbers compared to non-climbers, and Saul et al. (1) emphasized the importance of aerobic forearm capacities and hand grip endurance. In addition to these factors, mental endurance, and anthropometric factors explained 77% of climbing ability in a study conducted by Magiera et al. (5). Laffaye et al. (4) found that 64% of the total variance in climbing ability could be explained by trainable variables such as upper limb and finger strength and anthropometric variables such as body composition and biacromial breadth. Trainable variables including upper limb and finger strength, lower limb power, as well as shoulder and knee flexibility according to Mermier et al. (6) explained 58.9% of the total variance in climbing ability. In addition, Grant et al. (3) found greater shoulder girdle endurance and hip flexibility in advanced climbers compared to both recreational climbers and non-climbers, and Saul et al. (1) emphasized the importance of postural stability and selected anthropometric factors such as a low body fat percentage and large forearm volume for climbing ability. Furthermore, they described climbers as having high mental endurance and low in tension, depression, anger, and confusion. Although differences in the weighting of the various factors were found between the different climbing disciplines (4, 7–10), the overall requirements for the disciplines formally correspond to the same categories.

Based on the findings on performance requirements in climbing, research in the field of training to improve climbing ability has been increasing. Performance diagnostics in climbing have therefore become increasingly important in order to determine performance deficits and measure training effects. However, the diagnostic tests lack consistency and only few studies include quality assessments for the tests used.

Within this review climbing performance as an empirical indicator is defined as a measurable variable represented by a test score. Climbing ability on the other hand is defined as the potential to achieve high climbing performance and refers to the theoretical construct which all variables are set in relation to. It is assessed individually through self-reporting of ability level with the help of (inter-)national grading systems in each study.

The most important criteria for test quality are validity and reliability. Validity “refers to the degree to which evidence and theory support the interpretations of test scores for proposed uses of tests” (11). Therefore, validity is not a feature of the test itself but rather of test interpretation. Different subcategories of validity can be distinguished. This review especially addresses construct and criterion validity as two closely related concepts. Construct validity refers to “the concept or characteristic that a test is designed to measure” (11). Regarding physical climbing diagnostics, test interpretations have high construct validity when there is evidence that test scores represent theoretical components of climbing ability or tests show a predefined/theoretical factor structure; for example, correlation with self-estimated climbing ability or Cohen's d as a measure of the difference between different ability groups is an indicator of construct validity. Criterion validity refers to the correlation between a test score and a measured criterion variable (11), which in this case is climbing performance. For example, Spearman's and Pearson's correlation coefficients between test scores and climbing performance were used for assessing criterion validity.

High validity requires high reliability. Reliability refers to measurement consistency or in other words an acceptable measurement error allowing effective practical use of the measurement (12). In this review we will differentiate between intra-session and inter-session reliability referring to measurement consistency within and between sessions, respectively. The prerequisites for measurement consistency are a high conformity across raters (inter-rater reliability) and within the ratings of a single rater (intra-rater reliability) (12). Reliability can be measured with different tools. In this review intra-class correlation coefficient (ICC), concordance correlation coefficient (CCC), Spearman's and Pearson's correlation coefficient were considered. In addition, the coefficient of variation (CV) and the standard error of mean (SEM) were considered as indicators of reliability.

The heterogeneity of the tests and the lack of reports on test quality can lead to problems when comparing the effects of different training interventions (13). In addition, researchers, coaches, and athletes find it difficult to select appropriate tests for their diagnostic test batteries. Approaches to create and validate a sport-specific test battery for climbing revealed low construct validity in relation to climbing ability for most of the selected tests, as well as tests that only allowed differentiation between specific performance groups (14, 15).

The aim of this review was therefore to give an overview of the tests for performance, strength, endurance and flexibility in climbing and their quality in order to identify strengths and weaknesses of existing tests and to support more homogeneous test batteries for future performance assessments and quantification of training effects.

## 2. Methods

### 2.1. Search strategy and data sources

The literature research and analysis followed the Preferred Reporting Items for Systematic Review and Meta-Analyses (PRISMA) guidelines (16), and the study selection process described by Meline (17).

A systematic literature search on PubMed and SPORTDiscus was performed in June 2022. Additionally, the retrieved articles were manually searched for additional articles possibly fulfilling the inclusion criteria. The search was conducted with the following terms: “performance”, “strength”, “force”, “power”, “endurance”, “aerobic capacity”, “anaerobic capacity”, “flexibility”, “agility”, “boulder”, “climb”, “assess”, “measur”, “hand dynamomet”, “test”, “diagnostic”. The wildcard symbol “*” and Boolean operators (OR and AND) were included to maximize and optimize the search.

### 2.2. Inclusion and exclusion criteria

To be included, studies had to be published in either English or in German. All studies including detailed information on at least one quantitative method of testing or measuring forms a) strength, b) endurance, c) flexibility, or d) performance in climbing and/or bouldering were included into the review. As we were interested in the quality of the tests in climbing and bouldering, only studies examining a representative sample of human boulderers and/or climbers were considered. In addition, studies had to contain detailed information on the subjects (age, sex, discipline, and experience) and report climbing ability levels using a recognized national or international scale. Randomized-controlled, cohort-, cross-over-, intervention- and case(-control) studies were included into the review. Publication types included were journal publications, dissertations, abstracts, and articles published in conference proceedings. Qualitative, explorative, and anecdotal research were not included into the review as they do not allow a quantitative analysis of the tests and measurements used.

### 2.3. Data extraction

The data on the diagnostic tests was extracted using a standardized form including sample characteristics (sample size, sex, discipline, ability level, age, experience, health), and variables related to each test and measuring method reported in the studies (test design, exercise, device, measured value, unit, reliability, validity). Reported grades for climbing and bouldering performance were standardized according to the International Rock Climbing Research Association (IRCRA) reporting scale (18).

### 2.4. Test classification and quality analysis

In a next step, the tests were sorted according to the exercises the subjects had to perform. For example, all tests in which the subjects had to do pull-ups were grouped together. Subsequently, the tests within each test group were classified according to a) measured values, b) exercise intensity (edge depth, percentage of MVC), c) exercise duration (time under tension/work time), d) involved body parts (fingers, upper limbs, lower limbs, core), and e) test execution (continuous or intermittent; isometric or dynamic). The quality of all tests within each test group in combination with sex and ability level of the respective subjects was then sorted according to the respective classification in a respective table. In a last step, the reliability and validity ranges for each test group were determined and summarized depending on the muscle groups (upper limbs, lower limbs, core, fingers) and the variable tested (strength, endurance, flexibility, or climbing performance). Regarding strength, a distinction was made between maximum strength, explosive strength (power), and strength endurance. In addition, strength endurance was divided into three subcategories. High intensity strength endurance was defined as maximum strength endurance (intensity: 90%–100%), submaximal strength endurance was defined as muscular endurance (intensity: 40%–80%) and explosive contractions to failure were defined as explosive strength endurance (intensity: 30%–60%, maximal power or rate of force development). Furthermore, static and dynamic flexibility as well as anaerobic and aerobic endurance were distinguished.

Correlations, effect sizes, and coefficients were rated as proposed by Akoglu (19), Koo and Li (20), Cohen (21), and Reed et al. (22) (Table 1). To facilitate understanding the different scales were transformed to a common three-point scale: low—middle-sized—high. In addition, we transformed r^2^ values to r values in order to apply the three categories. SEM was evaluated for each study individually according to the recommendations by Denegar and Ball (23).

**Table 1:** Ratings of correlations, effect sizes, and coefficients.

Parameter	Grading	
ICC	<0.5	– Poor
	0.5**–**0.75	– Moderate
	0.76**–**0.89	– Good
	≥0.9	
CCC	<0.90	– Poor
	0.9**–**0.95	– Moderate
	0.96**–**0.98	– Substantial
	≥0.99	– Almost perfect
Pearson's and Spearman's r	0	– No correlation
	0.1**–**0.3	– Weak
	0.4**–**0.6	– Moderate
	0.7**–**0.9	– Strong
	1	– Excellent
Cohen's d	< 0.2	– Negligible
	0.2**-**<0.5	– Small
	0.5**-**<0.8	– Medium
	≥0.8	– Large
CV	≤ 20%	– Acceptable
	>20%	– Poor
**Own terminology**		
No correlation, negligible	– No correlation	
Poor, weak, small	– Low	
Moderate, medium	– Middle-sized	
Good, substantial, strong, large	– High	
Excellent, almost perfect	– Very high	
Acceptable	– Acceptable	
Poor	– Poor	

ICC, intraclass correlation coefficient; CCC, concordance correlation coefficient.

## 3. Results

### 3.1. Study selection and characteristics

**Figure 2:**
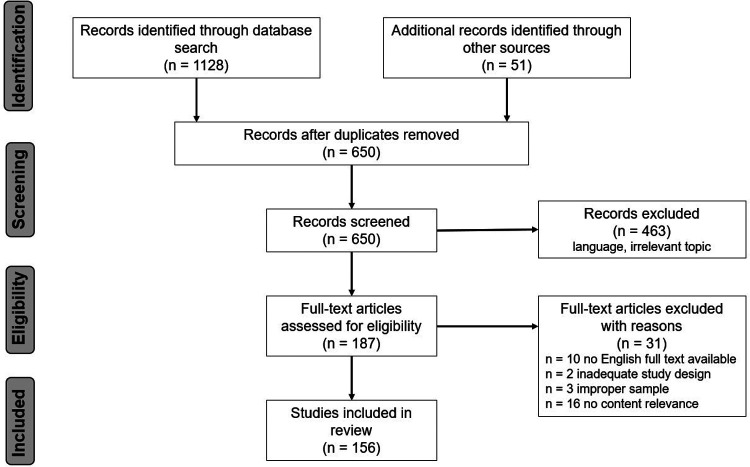
PRISMA flow diagram.

A total of 1,128 studies were identified by searching PubMed and Sport DISCUS. By manually searching the reference lists of these articles, 51 further studies were identified. After the removal of the duplicates and 463 studies, which did not fulfill the content or language requirements, 187 full texts were assessed for eligibility. Due to different reasons such as insufficient content relevance or inadequate study design, 31 studies were excluded. In the case of six studies (24–29), the abstract was found to provide sufficient information to include the conducted tests into the study. Ultimately, 156 studies were included in the review (Figure 2).

**Figure 3:**
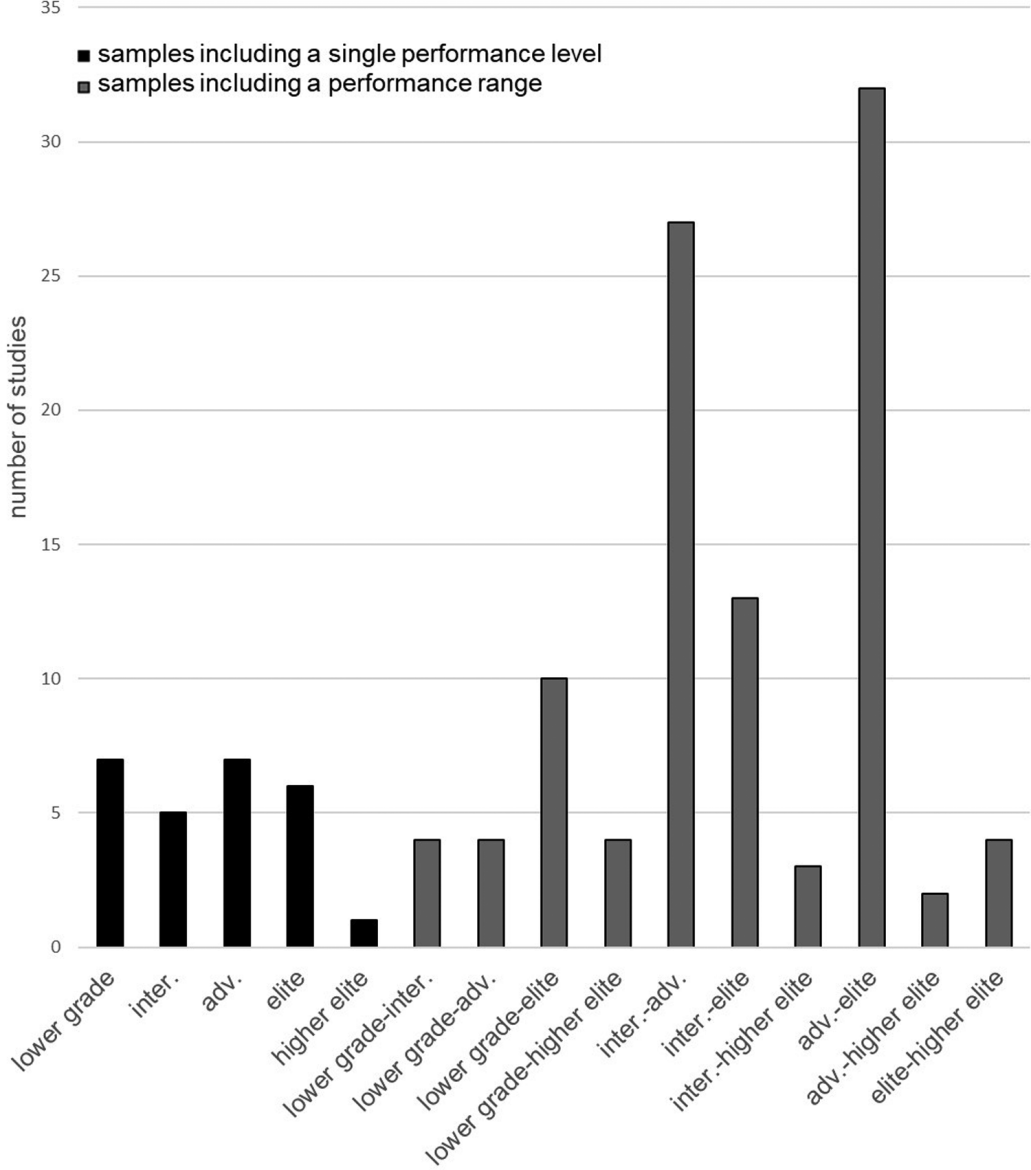
Overview of the samples in the included studies.

Figure 3 shows the climbing ability of the various samples investigated in the studies according to the IRCRA reporting scale (18). It also gives an overview of the number of studies focusing on similar sample characteristics regarding climbing ability. While 32 studies included advanced to elite climbers, 27 focused on intermediate to advanced athletes. Only one study exclusively included higher elite climbers while four studies each included lower grade to higher elite and elite to higher elite climbers. Three studies focused on intermediate to higher elite and two on advanced to higher elite climbers. Thirteen and ten studies dealt with climbers from the intermediate and lower levels to the elite, respectively. In seven, five, and six studies only lower grade, advanced, intermediate and elite climbers were considered, respectively. Four studies each included lower grade to intermediate and lower grade to advanced climbers. Nineteen studies did not report the climbing ability of their sample.

**Figure 4:**
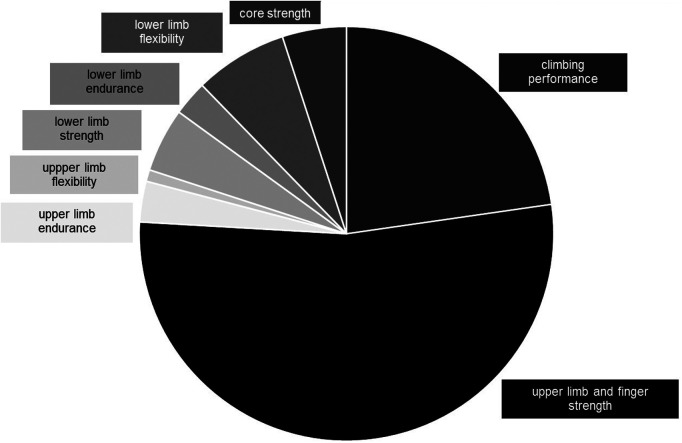
Overview of the distribution of tests within the identified studies.

Within the studies a total of 429 strength, endurance, flexibility and performance tests were identified. 53% of the studies included upper limb and finger strength tests, 23% included climbing performance tests, 7% included lower limb flexibility tests, 5% each included core strength and lower limb strength tests, 3% each included upper and lower limb endurance tests, and 1% included upper limb flexibility tests (Figure 4).

### 3.2. Findings

A total of 66 test groups were identified. For many of these, many different ways of implementation of the respective tests were found. Seven tests measuring tactics, technique, hip flexibility, core strength endurance, and upper limb and finger strength endurance and maximum strength, were not included into the analysis as the studies did not include enough information on the test execution (5, 30, 31). One test examining route reading skills conducted by two studies (15, 32) was also not included into the analysis as it does not relate to physical climbing skills. One study conducted a 100-metre run (33). This test was also not included into the analysis due to its lack of specificity.

The tables presenting the quality of all tests within a test group in combination with sex and ability grading of the respective subjects can be found in the supplementary material (Supplementary Material Tables S1–66). Tables 2–9 sum up the reliability and validity ranges for each test group.

**Table 2:** Reliability and validity measures for climbing and bouldering performance tests.

Bouldering/climbing	Measured variable	Reliability	Construct validity (correlation with self-reported climbing ability)
Repeated ascent of 1 boulder (34, 35)	*Bouldering/climbing E*	Inter session: *r* = .99 (34)	*r* = .87 (34)
Boulder in a circuit (26, 36, 37)	*Bouldering/climbing E*	–	*r* = -.84 –.43 (37); *r* = .88 (37)
Boulder traverse (38, 39)	*Bouldering/climbing E*	–	*r* = .52 –.94 (38)
Treadwall climbing (40–47)	*Bouldering/climbing E*	Inter-session: *r* = .99 (40)	*r* = .81 –.91 (41, 42); *r* = -.66—−0.28 (43); d = .02–1.46 (41)
Top-rope and lead climbing combined (28, 48)	*Climbing E*	–	–
Outdoor climbing (49)	*Climbing E*	–	–
Rock over climbing test (50)	*Bouldering/climbing ability*	Inter-session: ICC = .90 (50)	–
Bouldering (7, 51–55)	*Bouldering ability*	–	*r* = -.47 –.39 (52)
Top-rope climbing (24, 56–71)	*Climbing E/ability/speed*	Inter-session: ICC = .97 (59); *r* = 0.10–0.48 (62); d = 0.69 (62)	–
	*Climbing kinematics*	Inter-rater: *r* = .88 (70)	*r* = .99 (68)
	*Climbing dynamics*	–	–
Lead climbing (6, 7, 28, 72–77)	*Climbing E/ability*	Inter-session: *r* = .81 (6)	*r* = .45 –.69 (73); *r* = .77 (6)
	*Climbing kinematics*	Inter session: *r* = .71 –.92 (74) Inter-rater: r > .81 (74)	–
	*Climbing dynamics*	–	–
Speed climbing start (78)	*Speed climbing dynamics*	–	–
1 speed climbing run (33, 79)	*Speed climbing ability*	–	–

E, Endurance.

**Table 3:** Reliability and validity measures for upper limb and finger strength tests.

Upper limb and finger strength	Measured variable	Reliability	Construct validity (correlation with self-reported climbing ability)
Dead hang (14, 15, 25, 27, 31–34, 54, 62–64, 80–93)	*Finger iso. ME*	Inter-session: ICC = .13—>.99 (14, 81–83), CV% = 18.0 (14), CV% = 23.4–29.9 (83), CV% = 12.8 (82)	*r* = -.26 –.87 (27, 62, 81, 83, 85, 87, 88); *r* = .90 –.93 (84)
	*Finger iso. inter. ME*	Inter-session: ICC = .97 (80); *r *= .86 (34)	–
	*Finger iso. MS*	inter-session: ICC = .93 –.99 (81, 82), CV% = 7.8 (82)	*r* = .58 –.84 (81, 83)
	*Finger iso. ME/MS*	–	–
Bent arm hang (3, 6, 14, 15, 30, 31, 46, 54, 63, 64, 82, 84, 94–99)	*Upper limb + finger iso. SE/MS*	Inter-session: ICC = .89, CV% = 15.0 (14); *r* = .97 –.99 (15)	*r* = .23—>.80 (15, 84, 94, 99) *r* = .77 (6)^+^
Pull-up (3, 7, 14, 31, 33, 46, 53, 54, 83, 85, 87, 89, 94, 95, 97, 100–106)	*Upper limb con. MS*	Inter-session: ICC = .84 –.99, CV% = 1.0–6.62 (100)	–
	*Upper limb ESE*	–	–
	*Upper limb con.-ecc. ME*	Intra-session: ICC = .97, CV% = 14.0 (14) Inter-session: ICC = .96 –.99 (14, 102), CV% = 14.0 (14)	*r* = .08 –.72 (83, 94)
	*Upper limb + finger con.-ecc. MSE*	–	–
	*Upper limb + finger iso. MS*	–	–
	*Upper limb iso. ES and Upper limb + finger MS*	Intra-session: ICC = .88 –.99, CV% = 9.1–12.9 (103)	*r* = .61 –.77 (85)
Pinch a dynamometer (3, 6, 24, 94, 95, 97, 102, 107–110)	*Pinch/pincer iso. MS*	Intra-session: r > .99 (108)	*r* = .22 –.59 (94, 109, 110); *r* = .77 (6, 95); CCC = .99 (107) *r* = .77 (6)^+^ (performance on multiple routes combined)
Grip a hand dynamometer (3, 4, 6, 24, 34, 38, 45–47, 49, 56, 57, 61, 62, 65, 73, 75, 80, 83–85, 94–97, 99, 109, 111–129)	*Hand iso. MS*	Intra-session: ICC≤.97 (4, 117), CV% = 3.2 (4) Inter-session: ICC = .91 –.98 (80, 112) Intra-rater: ICC = .88 (118)	*r* = -.96 –.72 (24, 73, 94, 95, 99, 121, 123) *r* = .77 (6)^+^; *r* = -.97—-.88 (24)^+^; *r* = .11 (121)^+^
	*Hand iso. ES*	–	–
	*Hand iso. MS + ES*	Intra-session: ICC = .94 –.99, CV% = 3.79–22.96 (115) Inter-session: ICC = .83 –.98, CV% = 4–6 (119)	–
	*Hand iso. ME*	–	*r* = .76 (6)^+^
	*Hand inter. iso. MSE*	Inter-session: ICC = .93, CV% = 3.2 (4)	*r* = -.60 (62)
Apply force on hold (2, 7–9, 14, 15, 24, 29, 32, 35, 38, 41, 47, 54, 90, 93, 96, 99, 101, 105, 106, 109, 110, 117, 122, 130–155)	*Finger iso. ES + MS*	Intra-session: ICC = .21 –.99 (130, 140), CV% = 2.64–28.34 (140) Inter-session: ICC = .40 –.94 (130); 0.60 < *r* < 0.80 (140)	*r* = .65-.76 (130)
	*Finger iso. (inter.) ME*	Intra-session (sus + inter): ICC = .85 –.92 (138) Inter session (inter): ICC = .29–91 (130, 142), CV%<2.5 (142)	Sus: *r* = -.26 –.72 (110, 138, 156); d = .44–1.47 (41); *r* = .76 (41) Inter: *r* = —27 –.19 (156); d = .07 –.33 (41); *r* = .65 (41)
	*Finger iso. (inter.) MSE/CF*	Intra-session (sus): ICC = .85 –.92 (138) Inter session (sus): ICC = .92 –.94 (130); (inter):.87 –.96 (132)	Sus: *r* = 80 –.82 (138, 156); *r* = .65–73 (130) Inter: *r* = .60 (99); *r* = .51 –.78 (132)
	*Finger iso. (inter.) MS*	Inter-session (sus): ICC = .88 –.92 (96, 130, 144), CV% = 2.2 (96); *r* = .88 –.99 (136, 143) Intra-session (sus): ICC = .97 –.98 (117); *r* = .88 –.95 (136, 138); Cronbach's alpha = .99 (110)	Sus: *r* = -.96 –.81 (2, 24, 99, 110, 136, 138, 144, 156); *r* = .04 –.92 (41, 95, 130, 131, 134, 147) *r* = -.94—-.77 (24)^+^, *r* = .43 –.67 (131)^+^
	*Finger + wrist con.-ecc. MS*	–	*r* = .57 (133)
Power-slap test (4, 14, 31, 32, 53, 54, 112, 134, 157, 158)	*Upper limb con. ES*	Intra-session: ICC = .98, CV%<4.89 (157) Inter-session: ICC = .95 –.98 (14, 112, 157, 158), CV%<4.89 (157), CV%=7.0 (14)	*r* = .69 –.73 (14, 157, 158)
	*Upper limb con. ESE*	–	–
Medicine ball throw (31, 112)	*Upper limb ES*	Inter-session: ICC = .96 (112)	–
Elbow strength tests (159)	*Upper limb MS*	–	*r* = .51 –.63 (159)
Biceps strength test (95)	*Biceps MS*	–	*r* = .29 –.45 (95)
Shoulder strength test (6, 160)	*Shoulder con.-ecc. MS*	–	–
	*Shoulder con. MS*	–	*r* = 0.77 (6)^+^
Push-ups (31)	*Upper limb ESE*	–	–
Campus board performance (39, 53)	*Upper limb ESE*	–	–
Arm jump test (161)	*Upper limb (ecc.)-con. ES*	–	–
Bench press (4)	*Upper limb con. ES + MS*	–	–
Pull down (63, 64)	*Upper limb con.-ecc. MSE*	–	–
Traction test (139)	*Upper limb con. ES*	–	–
	*Upper limb con.-ecc. ME*	–	–

CF, critical force; MS, maximum strength; ME, muscular endurance; ES, explosive strength; MSE, maximum strength endurance; ESE, explosive strength endurance; iso., isometric; con., concentric; ecc., eccentric; sus, sustained contraction; inter, intermittent contraction; CV, coefficient of variation; +, criterion validity (correlation with climbing performance test scores).

**Table 4:** Reliability and validity measures for upper limb endurance tests.

Upper limb endurance	Measured variable	Reliability	Construct validity (correlation with self-reported climbing ability)
Rowing ergometry (162–164)	*Con.-ecc. E*	–	*r *= .85 (162)
	*Con. MS*	Inter-session: ICC = .79 –.85 (163)	*r *= .72 –.73 (163)
Arm crank ergometry (30, 49, 95, 165)	*Con.-ecc. E*	* *	*r *= .20 –.56 (95)

MS, maximum strength; E, endurance; con., concentric; ecc., eccentric.

**Table 5:** Reliability and validity measures for upper limb flexibility tests.

Upper limb flexibility	Measured variable	Reliability	Construct validity (correlation with self-reported climbing ability)
Shoulder flexibility test (6, 134)	*Shoulder active dynamic FLEX (overhead)*	–	–
Shoulder abduction and flexion (6)	*Shoulder active static FLEX (range of motion)*	–	*r *= .14 (6)^+^

FLEX, flexibility; ^+^, criterion validity (correlation with climbing performance test scores).

**Table 6:** Reliability and validity measures for lower limb strength tests.

Lower limb strength	Measured variable	Reliability	Construct validity (correlation with self-reported climbing ability)
Lower limb strength test (6)	*Con. MS*	–	*r *=0.77 (6)^+^
jump with high foot (15, 32)	*Con. ES*	Intra-session: *r *= .76–92 (15)	–
Counter movement jump (32, 47, 79, 134)	*Ecc.-con. ES*	–	*r *=.79 (79)
Squat jump (32, 47, 94)	*Con. ES*	–	*r *=.23 –.33 (94)
Standing long jump (31, 33)	*Ecc.-conc. ES*	–	–
Vertical jump (54)	*(Ecc.-)con. ES*	–	–
One legged squats (113)	*Con.-ecc. ME*	–	–

MS, maximum strength; ES, explosive strength; ME, muscular endurance; con., concentric; ecc., eccentric; ^+^, criterion validity (correlation with climbing performance test scores).

**Table 7:** Reliability and validity measures for lower limb endurance tests.

Lower limb endurance	Measured variable	Reliability	Construct validity (correlation with self-reported climbing ability)
**Treadmill running** (37, 41, 45, 95, 162)	*E*	–	d = .17 –.43 (41); *r* = .17-.28 (95), ns (162)
**Cycle ergometry** (6, 42, 166–168)	*E*	–	–

E, Endurance; ns, non-significant.

**Table 8:** Reliability and validity measures for lower limb flexibility tests.

Lower limb flexibility	Measured variable	Reliability	Construct validity (correlation with self-reported climbing ability)
**Sit and reach** (3, 47, 95, 97, 114, 169)	*Low back + hamstring Active static FLEX*	Inter-session: ICC = .97 (169)	*r *= 0.17–0.42 (95)
**Lateral foot reach** (169)	*Hip active static FLEX*	Inter-session: ICC = .93 (169)	*r *= .24 –.30 (169)
**Grant foot raise** (3, 95, 97, 110, 169, 170)	*Hip active static FLEX*	Inter-session: ICC = .90 –.93 (169)	*r *= .20 –.34 (110, 169); *r *= .26 –.49 (95)
**Climbing specific foot raise** (14, 15, 32, 169)	*Hip active static FLEX*	Inter-session: ICC = .89 (169); *r *= .95 –.99 (15)	*r *= .53 –.95 (14, 15, 169)
**Hip abduction test** (6, 131)	*Hip active static FLEX*	–	*r *= .14 (6)^+^
**Draga test** (170)	*Hip active static FLEX*	–	–
**Hip slide test** (134)	*Hip active static FLEX*	–	–
**Foot loading flexibility test** (169)	*Hip active static FLEX/Climbing ability*	Inter-session: ICC = .96 (169)	*r *= .56 –.65 (169)
**Asymmetry in reach test** (113)	*Hip active static FLEX/Climbing ability*	Intra-session: ICC = .89—>.99, CV% = 1.31–35.20, SEM%=.09 –.61 (113), inter-session: ICC = .87 -.96, CV% = 4.96–41.98, SEM% = .07–1.57 (113)	–
**Froggies** (5, 30)	*Hip passive static FLEX*	–	–
**Straddle test** (3, 95, 97, 134, 170)	*Hip + lower limb passive Static FLEX*	–	*r *= -.48—-.41 (170); *r *= .16 –.57 (95)
**Hip rotation and flexion** (131)	*Hip active FLEX*	–	–
**Leg flexion** (131)	*Lower limb active FLEX*	–	–

FLEX, flexibility; CV, coefficient of variation; SEM, standard error of mean; ^+^, criterion validity (correlation with climbing performance test scores).

**Table 9:** Reliability and validity measures for core strength tests.

Core strength	Measured variable	Reliability	Construct validity (correlation with self-reported climbing ability)
**Super-man** (86)	*Con.-ecc. MS*	Inter-session: ICC = 0.87 (86)	–
**Momentum absorption** (15, 32)	*Con. MS*	–	*r* = -.01 –.31 (15)
**Core rotation test** (86)	*Con. MS*	–	–
**Body lock off** (86)	*Iso. SE*	Inter-session: ICC = .79 (86)	–
**Plank** (14)	*Iso. SE*	–	–
**Sorensen test** (4, 96)	*Iso. SE*	–	–
**Kraus Weber test battery** (96)	*Iso. SE*	–	–
**Sit-ups** (31)	*Con.-ecc- SE*	–	–
**Curl-ups** (3, 97, 114)	*Con.-ecc- SE*	–	–
**Fishing kicks** (15, 32), (86)	*Con.-ecc. SE*	Inter-session: ICC = 0.91 (86)	*r* = -.42—-.12 (15)
**Leg raise** (14, 95, 96)	*Core + lower leg iso. SE*	–	*r* = .30 –.45 (95)

MS, maximum strength; ME, muscular endurance; iso., isometric; con., concentric; ecc, eccentric.

#### 3.2.1. Climbing performance

Climbing performance tests (Table 2) take on a special position. This is due to the fact that the measured value through the following tests highly depends on the design of the climbing wall:

• *P*epeated ascent of one boulder
• *B*ouldering in a circuit
• *T*readwall climbing
• *T*raverse bouldering
• *T*op-rope and/or lead climbing
• *B*ouldering

Other tests work with a standardized wall design:

• *P*ock over climbing test
• ne speed climbing run
• Speed climbing start

Medernach et al. (34) reported a high inter-session reliability and a high correlation between climbing ability and the test results for the repeated ascent of one boulder. Deyhle et al. (36) asked their subjects to boulder in a circuit following the rhythm of a metronome until exhaustion while Limmer et al. (26) only state that their subjects had to do some lap climbing. Both do not report any test quality data.

Both Michailov et al. (38) and Sas-Nowosielski et al. (39) tried to assess climbing performance through a boulder traverse. Sas-Nowosielski et al. (39) included a hard traverse with crimp and half-crimp holds and an easy traverse with pinch holds which the subjects had to climb back and forth until exhaustion. Michailov et al. (38) also included two routes, one of which had holds with an inclined contact surface and the other holds with a horizontal contact surface. Sas-Nowosielski et al. (39) did not provide test quality data. Michailov et al. (38) on the other hand report a high correlation between time to failure and climbing ability for the hard traverse and a middle-sized correlation for the easy traverse.

Treadwall climbing was used as a diagnostic tool by Schoeffl et al. (40) who report a high inter-session reliability. They had asked their subjects to climb a given route on a treadwall at constant speed and inclination until exhaustion. Studies by Baláš et al. (41) and Limonta et al. (42) report high to very high correlations between treadwall peak angle, systemic V˙O_2_ from submaximal climbing, local muscle tissue oxygen saturation (StO_2_) from submaximal climbing, and muscle oxygenation breakpoint and climbing ability. Baláš et al. (41) conducted a test in which the subjects started at an inclination of 0° and had to climb until exhaustion, with the inclination of the treadwall increasing by 5° every minute. They also found low to high differences between intermediate and elite climbers regarding Treadwall peak angle. In another study Baláš et al. (43) conducted a similar test starting at +6° and an increasing angle of inclination of -3° per minute to identify the critical angle and multiple exhaustive tests at various fixed angles to estimate the critical angle. While the peak angle reached during the incremental test showed middle-sized correlations to both climbing and bouldering ability, the estimated critical angle showed only low correlations to climbing and bouldering ability.

Limonta et al. (42) conducted a discontinuous test in which the subjects started with 5 min of baseline measurements followed by the same two workloads, controlled over the speed, for all participants and three more workloads, each lasting 4 min, with 5 min of rest in between according to individual cardiorespiratory response to reach peak aerobic power in 5 workloads. Booth et al. (44) conducted a test with a similar protocol including three trials at increasing velocity and 20 min rest between the trials. Fryer et al. (45), Potter et al. (46), Booth et al. (44), and España -Romero et al. (47) do not report quality data. While Fryer et al. (45) and España -Romero et al. both conducted an incremental test, Fryer et al. (45) gradually increased the inclination of the wall, with the subjects starting at different angles according to their climbing ability, whereas España-Romero et al. (47) gradually increased the climbing speed. Potter et al. (46) asked their subjects to do three self-paced climbs on the treadwall until exhaustion. Baláš et al. (37) measured mean oxygen consumption and heart rate during bouldering in a circuit until exhaustion with an increase in wall inclination by 10° every three minutes. They reported a high negative correlation between mean oxygen consumption and climbing ability and a middle-sized negative correlation between heart rate and climbing ability. Additionally, they found a high correlation between climbing ability and the wall inclination at the moment of exhaustion. Deyhle at al (36). and Limmer et al. (26) did not provide any quality data.

Top-rope climbing was used in several different ways to assess multiple different factors of climbing performance. Jurrens (56) and Kingsley (57) provided 12 climbing routes with various levels of difficulty and awarded points for each handhold reached by the participants. Barton (58), and McNamee and Steffen (59) conducted a similar test. The subjects started with a route of their choice. If they reached the top, they continued with the next more difficult route. If they did not reach the top, they continued with an easier route. At the end, the highest grip reached on the most difficult route was counted if the next easier route was topped. Fraser (60) determined the highest hold achieved on the most difficult route attempted. Heyman et al. (61) asked their subjects to climb a route twice to volitional exhaustion. If they reached the top they immediately started again from the bottom. The test conducted by Limmer et al. (62) is very similar. Their subjects were asked to climb a route as often as possible with no rest in between the attempts. Hermans et al. (63) and Hermans (64) assessed the point of failure of each subject in a route they were asked to climb to failure once. While the participants in the study of Valenzuela et al. (65) had to cover as much distance as possible in one route within two minutes, Bertuzzi et al. (66) assessed the distance climbed up and down a route in three minutes. Sanchez et al. (67), Seifert et al. (68, 69), Jones et al. (70), and Mitchell et al. (24) assessed different factors while their subjects climbed one to three routes at their own pace. The participants of a study by Baláš et al. (71) however, climbed a route up and down twice at a given pace. Vertical reaction force under each foot was assessed. McNamee and Steffen (59) reported a very high inter-session reliability for their test for climbing ability. Limmer et al. (62) reported low to middle-sized correlation between test trails for time to failure and post activity lactate levels. Additionally, they reported middle-sized differences for post activity lactate levels between trials. Jones et al. (70) assessed climbing kinematics through the score on an observer scale and found a high correlation between the ratings by different experts. No further quality data were reported on top-rope climbing tests.

Lead climbing was also used as a diagnostic tool to assess climbing ability, endurance, kinematics, and dynamics. Multiple authors (6, 7, 72–74) have asked their subjects to climb one or two routes until failure. Magiera et al. (75) have assessed mean climbing difficulty through the performance of the subjects on multiple routes. Assessing performance during a competition is a tool used by Sanchez et al. (76) and Fuss et al. (77). Magiera et al. (75) reported a high correlation between the climber's performance on different routes. Middle-sized to high correlations were found by Taylor et al. (74) for the expert ratings between sessions and high correlations between the ratings of various experts regarding technical and tactical factors. The only data on test validity for lead climbing are reported by Gajewski et al. (73) and Mermier et al. (6). The former found a middle-sized correlation between climbing ability and post-exercise lactate recovery. The latter report a high correlation between the trainable variable in climbing, including climbing rating, and multiple power, and flexibility measurements, and climbing ability. Few studies assessed climbing endurance through a mixture of top-rope and lead climbing, or outdoor climbing, but did not provide data on the quality of the tests (28, 48, 49).

Brent et al. (50) have tried to assess bouldering or climbing ability through a complex test called the rock over climbing test. They reported a high inter-session reliability but did not provide any data on the correlation with climbing ability.

Numerous studies have investigated bouldering ability through bouldering itself, using different approaches. White and Olsen (51) conducted a competition-like bouldering test with five 5 boulder problems for which the participants had six minutes each to solve and another six minutes rest in between. Zemtsova and Vavaev (52) observed the performance of their participants at the world championships 2018 in Innsbruck and 2019 in Hachioji including five boulder problems. The participants of the study by Frauman had to solve three boulder problems within five minutes each and five minutes rest in between. Stien et al. (7, 53) included three and four boulder problems respectively and gave the subjects four minutes to solve each of them and a three-minute rest between the boulder problems. Nichols et al. (54) also included three boulder problems. The only study reporting quality data were Zemtsova and Vavaev (52). They report middle-sized negative to middle-sized positive correlations between the test outcomes and climbing ability for multiple factors assessed (number of attempts per top and zone, number of grips, attempt time, recovery time, climbing time, and viewing time).

Speed climbing ability and speed climbing dynamics were assessed through the time taken for one speed climbing run (33, 79) and the directions of the mean forces during the speed climbing start (78). All three studies did not provide any information on the reliability of the tests or the correlation of their outcomes with (speed-) climbing ability.

In summary, climbing performance was assessed through nine different tests differentiating between climbing endurance, ability, kinematics, and dynamics. No study reported both reliability and validity data for any of the tests. However, the repeated ascent of one boulder, treadwall climbing, the rock over climbing tests, and top-rope climbing were shown to be highly reliable. The highest correlation with climbing ability was reported for the repeated ascent of one boulder.

#### 3.2.2. Upper limb and finger strength

The following tests were used to assess upper limb and finger strength (Table 3):

• Dead hang
• *Β*ent arm hang
• Pull-up
• Push-up
• Campus board performance test
• *Β*ench press
• Pull down
• *T*raction test
• *M*edicine ball throw
• Shoulder strength tests
• *B*iceps strength test
• *E*lbow strength tests
• Power-slap test
• *A*rm jump test
• Gripping a dynamometer
• *A*pplying force on a hold
• Pinching a dynamometer

The dead hang was used to assess finger isometric muscular endurance in continuous and intermittent tests. It was also used to assess finger isometric maximum strength by holding maximum weight for 3–7 s. A mixture of muscular endurance and maximum strength was assessed by hanging to failure on very narrow edges or the one-arm dead hang. The implementation of the test varies substantially in terms of the grip type, the edge depth and the grip width used. High to very high inter-session reliability is reported for the tests on finger isometric intermittent muscular endurance and finger isometric maximum strength. Medernach et al. (34, 80) worked with a hang to rest ratio of 8:4 s on a 30 mm edge with open crimp. Bergua et al. (81) used a 40 mm edge and let their participants (advanced to elite males and females) choose between open- and half crimp, whereas López-Rivera and Gonzáles-Badillo (82) used a 15 mm edge when testing elite climbers and allowed half crimp only. The reliability of the dead hang tests to assess sustained isometric muscular endurance of the fingers is reported to be very high by Bergua et al. (14 mm or 25 mm edge with open- or half crimp) (81), Draper et al. (14) (30 mm edge with self-chosen grip), and López-Rivera and Gonzáles-Badillo (11 mm edge with half crimp) (82). Ozimek et al. (83) used a metal bar instead of an edge and reported a low to high inter-session reliability for elite male climbers. No reliability data is provided for tests combining muscular endurance and maximum finger strength. Validity data is reported for the sustained muscular endurance tests. The correlations between the test results and climbing ability cover a wide range. Bergua et al. (81) report high negative correlations for the minimum edge depth the participants could hang from for 40 s. Baláš et al. (84) and Kitaoka et al. (27) report high to very high positive correlations for maximum hangtime and post exercise lactate concentrations, respectively. Middle-sized to high correlations are reported between finger isometric maximum strength test results and climbing ability.

Like the dead hang, the bent arm hang was implemented with various grip types, edge depths and shoulder widths. Time to failure was assessed during a unilateral or a bilateral bent arm hang. Augste et al. (15) also assessed maximum weight held for 3 s in a unilateral bent arm hang. Thus, through different implementations, the bent arm hang can be used to assess upper limb isometric muscular endurance and maximum strength. If small holds are used, finger isometric maximum strength or muscular endurance also play a role in this test. Studies providing data on inter-session reliability, report very high ratings, including acceptable CV values, for the test design used in the IRCRA test-battery (14) and very high correlations between sessions for the maximum weight held for 3s in a one arm bent arm hang (15). Low to high correlations between test results and climbing ability were reported. Additionally, Mermier et al. (6) report a high correlation between the strength and the endurance component, including other strength and endurance tests, and climbing performance tested on multiple routes.

The pull-up was used to assess upper limb explosive strength (endurance) (33, 100, 101) and muscular endurance (14, 83, 94, 102). Furthermore, it was used to measure upper limb and finger maximum strength (endurance) (31). The isometric pull-up was implemented to assess upper limb isometric explosive strength (85, 103) as well as upper limb maximum strength and finger maximum strength (if small holds were used). Inter- and intra-session reliability measures, ranging between high and very high, were reported for multiple different pull-up variations (14, 102, 103, 111). Muscular endurance measures through the number of pull-ups performed show no to middle-sized correlation to climbing ability (83, 94). Middle-sized correlations were also found for peak force, and rate of force development (RFD) measured during an isometric pull-up by Vereide et al. (85).

Multiple tests such as push-ups, campus board performance, bench press, pull down and a traction test were used to assess upper limb explosive strength (endurance) and maximum strength (endurance). However, no quality data on any of these tests were reported.

Upper limb explosive strength was also assessed by measuring the maximum distance of a medicine ball throw. While no data on the correlation of the test measures with climbing ability were reported, Cochrane and Hawke (112) report a very high inter-session reliability.

For a test implemented by Mermier et al. (6) to assess shoulder concentric maximum strength, no quality data are being reported, except for a high correlation between shoulder strength and other strength and endurance tests, and climbing performance, measured on multiple routes. Wong (160), who tested eccentric and concentric strength of the shoulders did not provide any test quality data.

The only test implemented to specifically measure biceps maximum strength was conducted by MacKenzie et al. (95) who report a low to middle-sized correlation to climbing ability.

Augustsson et al. (159) were the only ones to examine elbow maximum strength in four tests including elbow flexion, extension, pronation, and supination. While no data on test reliability was reported, middle sized correlations to bouldering ability were reported.

The power-slap test is one of the most common tests used to assess upper limb explosive strength in climbers. Authors have measured the maximum height slapped with one hand or both hands at the same time and the highest rung reached and held for two seconds with one hand, respectively. Very high inter- and intra-session reliability were reported for the maximum height slapped with both one and two hands by multiple studies. However, no correlations with climbing ability were reported. The same is the case for quality data on the measurement of the fatigue index during multiple power-slaps to assess explosive strength endurance as conducted by Laffaye et al. (4).

Abreu et al. (161) asked their participants to perform an arm-jump test. This test is similar to the power slap test with both hands but instead of slapping the wall, the subjects are asked to reach and hold the highest possible rung. No quality data on measuring upper limb explosive strength through this test are reported.

Force parameters of the hand and fingers were assessed in multiple different ways. Three groups of tests were identified.

Firstly, hand dynamometers were used to measure hand force, which requires the use of the opposing thumb. Various different arm positions (shoulder flexion, elbow flexion, shoulder ab-/adduction), hand position (supination), and body positions (sitting or standing) were applied. In addition, the forearm was supported in some studies. Isometric maximum hand strength was assessed by measuring (mean) maximum force. Intra-rater reliability was reported to be high. In addition, intra-and inter-session reliability were reported to be very high. A very high negative correlation between the test results and top rope climbing time was reported by Mitchell et al. (24), while other authors have reported low to high positive correlations with top rope climbing time and self-reported climbing ability. Hand isometric explosive strength was assessed measuring RFD. No quality data are reported for these tests. Few studies measured both maximum strength and explosive strength during one test. Middle-sized correlations to climbing ability are reported for these tests and they show a very high intra- and inter-session reliability. Hand isometric muscular endurance was also tested through handheld dynamometry. Subjects were asked to maintain 50 or 80% of their MVC for as long as possible. While no data on the reliability of these tests are reported, Mermier et al. (6) report a high correlation between a group of strength and endurance tests including a handheld dynamometry test at 50% of MVC until exhaustion, and climbing ability. Moreover, hand intermittent isometric maximum strength endurance was assessed by measuring maximum force and fatigue index during repeated MVCs. A very high inter-session reliability and a middle-sized negative correlation with climbing ability are reported.

Secondly, finger strength without an opposing thumb was conducted by applying force on holds. Different hold types, hold depths, and various finger positions (slope crimp, half crimp, open crimp, pinch, jug, and sloper) were used. Furthermore, different arm positions (shoulder flexion, elbow flexion, shoulder ab-/adduction), and body positions (sitting, standing, hanging, crouching or leaning over a table) were applied. The forearm was supported during the tests in some studies. A combination of finger isometric explosive and maximum strength was assessed through one explosive MVC. Intra- and inter-session reliability were reported as low to very high. The test results explained 65% to 73% of the variability in climbing ability as reported by Michailov et al. (130). Finger isometric muscular endurance was assessed in both sustained and intermittent tests. Intra-session reliability for both variants was reported as high to very high. Inter-session reliability was only reported for the intermittent tests and ranged from low to very high. The correlation between the results from the sustained tests with climbing ability ranged from low negative to high positive. As reported by Baláš et al. (41), the test results were able to explain 56% of the variability in climbing ability. Furthermore, they found significant low to high differences between the test results of intermediate and advanced climbers. The correlation between the results from the intermittent tests with climbing ability ranged from low negative to low positive. As reported by Baláš et al. (41), the test results explained 43% of the variability in climbing ability. Furthermore, they found low but significant differences between the test results of intermediate and advanced climbers. In addition, Wall et al. (131) and Mitchell et al. (24) report high negative to middle-sized positive correlations with climbing performance on multiple routes and top-rope climbing time, respectively.

Finger maximum strength endurance and finger flexor critical force (132) were assessed through sustained and intermittent MVCs until failure, respectively. Intra-session reliability was reported to range between high and very high for the sustained tests. Inter-session reliability was reported to be very high for the sustained tests and high to very high for the intermittent tests. While high correlations to climbing ability were reported for the sustained tests, middle-sized correlations were reported for the intermittent tests. Tests assessing solely finger isometric maximum strength through intermittent and sustained contractions are reported to have a very high intra- and inter-session reliability. The correlation between the test results ranges from highly negative as reported by Mitchell et al. (24) to highly positive. One study by Schweizer and Furrer (133) assessed finger and wrist concentric-eccentric maximum strength with an especially designed apparatus. They reported a middle-sized correlation to climbing ability.

Thirdly, isometric pinch or pincer (only thumb and index finger) maximum strength were also assessed with a dynamometer. Depending on the study, different body positions were applied during the test. This includes shoulder and elbow flexion, body position (standing or sitting) and the fingers included into the pinch (I/II | I/III | I/II-III | I/II-IV | I/II-V). Studies report a high inter-session correlation and low to middle-sized correlation with climbing ability. Mundry et al. (107) report a high correlation with climbing ability. They had asked their participants to pinch a dynamometer while sitting on a chair with the upper arm leant on the thorax, the elbow at a 90° angle and the hand in a pronated position.

In summary, a total of sixteen tests for assessing upper limb and finger strength in climbing were identified. Several tests were used in multiple ways to assess different types of strength (maximum strength, muscular endurance, explosive strength, explosive/maximum strength endurance). Furthermore, test implementation varied greatly between the different studies. It was found that most tests still lack reliability assessment and validation. Few tests were reported to be highly reliable. This includes dead hang, bent arm hang, pull up, pinching a dynamometer, applying force on a hold, and the power-slap test. Due to the variety of test implementations, correlation ranges are large for most of the tests. Some of the highest correlations with climbing ability were reported for applying force on a hold or pinching a dynamometer.

#### 3.2.3. Upper limb endurance

Upper limb endurance was assessed by two tests (Table 4):

• Arm crank ergometry
• Rowing ergometry

Arm crank ergometry was used in several studies and different values such as maximum and average power, maximum force, maximum oxygen uptake, time to failure, and heart rate were measured. No data on the reliability of arm crank ergometry are reported and while Pires et al. (165) found significant differences between climbers and non-climbers regarding VO_2_-peak, the correlation with climbing ability was reported to be only low to middle-sized (95).

A high correlation with climbing ability was, however, found for maximum oxygen uptake during rowing ergometry by Michailov et al. (162). Marino et al. (163) used rowing ergometry to assess upper limb concentric maximum strength. The measurement through the one repetition maximum indicates a high reliability and a high correlation with climbing ability.

In summary, two tests were used to assess upper limb endurance in climbing but only few validity and reliability measures have been reported to this date.

#### 3.2.4. Upper limb flexibility

Upper limb flexibility was tested through two tests (Table 5):

• Shoulder abduction and flexion
• Shoulder flexibility test

Mermier et al. (6) assessed shoulder abduction and flexion through a test for the maximum active range of motion while standing with palms facing inward. Giles et al. (134) instead assessed the minimum distance between both hands gripping the same wooden stick that allowed for a full overhead rotation of the said stick without bending the arms. None of the two studies reported reliability measures. Validity measures were only reported by Mermier et al. (6) who found a low correlation between the flexibility component, including shoulder and lower limb flexibility, and climbing performance.

In summary, two tests assessing upper limb flexibility were implemented in climbing research, with only little data reported on test quality.

#### 3.2.5. Lower limb strength

Several tests used to assess lower limb strength were identified (Table 6):

• Squat jump
• Standing long jump
• Jump with high foot
• Counter movement jump (CMJ)
• Vertical jump
• One legged squat
• Unnamed lower limb strength test

While no study reported both reliability and validity data on any of the tests, Mermier et al. (6) report a high correlation between the strength and endurance component including the lower limb strength test and other strength and endurance tests, and climbing performance in climbers.

Augste et al. (15) specified a high intra-session and an unacceptable inter-session reliability for the test jump with high foot.

According to Augste et al. (32), the CMJ proved to be relevant to speed climbing and bouldering. In addition, Krawczyk et al. (79) found a high negative correlation between height and power for the CMJ and climbing time in speed climbing. Both España-Romero et al. (47) and Giles et al. (134), however, found no significant differences between climbers of different ability levels.

The squat jump was used in studies by España -Romero et al. (47), Augste et al. (15) and Arazi et al. (94). The latter could identify a low correlation between jump height and climbing ability in both males and females.

For the standing long jump used by Kozina et al. (33) and Stancović et al. (31), the vertical jump conducted by Nichols et al. (54), and the one legged squat applied by Čular et al. (113), no data on test quality is provided.

In summary, six different tests were used to measure lower limb strength in climbing research. While only very little quality data was reported, research points toward squat jump, and CMJ measurements as possible indicators of climbing-specific lower limb strength.

#### 3.2.6. Lower limb endurance

Lower limb endurance was tested through two tests (Table 7):

• Treadmill running
• Cycle ergometry

Only five studies used the cycle ergometer to conduct a discontinuous incremental test (42, 166–168) and the Wingate test protocol (6). Unfortunately, no data on the reliability or validity of the test were reported by Limonta et al. (42). However, the authors stated that they could not find any difference in maximum oxygen uptake between climbing and cycling. Mermier et al. (6) report a high correlation between the strength and endurance component including other upper- and lower limb endurance and strength test, and climbing performance.

MacKenzie et al. (95) found that aerobic capacity during a treadmill test with progressive inclination until volitional exhaustion shows a low correlation with climbing ability of both males and females. Michailov et al. (162) and Fryer et al. (45) on the other hand found no significant correlation between exhaustive treadmill running (continuous test with progressive speed and progressive speed and inclination respectively) and climbing performance. Baláš et al. (37) conducted a treadmill running test with progressive speed at constant inclination (5%) until exhaustion but did not report any reliability or validity data. Baláš et al. (41) found low differences between intermediate and advanced climbers during a treadmill running test with progressive inclination (%) to failure regarding time to failure, slope, tidal volume, respiratory exchange rate and heart rate.

In summary, two tests were established to measure lower limb endurance in climbing. No significant correlations were found between oxygen uptake during cycling and climbing, and treadmill running showed little or no correlation with climbing ability.

#### 3.2.7. Lower limb flexibility

Lower limb flexibility was assessed through multiple tests (Table 8). While some tests are also known in other sports, more climbing specific tests were developed:

• Sit and reach
• Lateral foot reach
• Grant foot raise
• Climbing specific foot raise
• Hip abduction test
• Draga test
• Hip slide test
• Foot loading flexibility test
• Asymmetry in reach test
• Froggies
• Straddle test
• Hip flexion and rotation
• Leg flexion

The sit and reach test as a test for low back and hamstring active static flexibility was used in multiple studies. Except for one study by Siegel et al. (114), who conducted the back saver sit and reach test, all studies conducted the sit and reach test with both legs. The only authors reporting reliability data are Draper et al. (169), who report a very high inter-session reliability. MacKenzie et al. (95) found a low and middle-sized correlation with climbing ability in males and females respectively.

Active static hip flexibility was assessed through several tests. Draper et al. (169) report a very high inter-session reliability but only a low correlation between test results and climbing ability for the lateral foot reach test.

A very high inter-session reliability is also reported for the Grant foot raise test by Draper et al. (169) for implementing the test both with and without lateral hip movement. However, only low to middle-sized correlations with climbing ability are reported for both males and females for all ways of implementation (with or without lateral hip movement and with a 23 cm or arm length distance to the wall).

The climbing specific foot raise test is very similar to the Grant foot raise test. The participants stand on footholds with their hands on a rung or handholds around head height. They then raise one foot as high as possible either with or without lateral rotation of the body to the wall. Draper et al. (169) found high inter-session reliability for the test without lateral rotation. Very high inter-session reliability was reported by Augste et al. (15). Middle-sized and high correlations were found between the test measures without and with rotation, respectively, and climbing ability.

Mermier et al. and Wall et al. (6, 131) conducted a hip abduction test. No test related quality data was reported. However, a low correlation between the flexibility component, including shoulder, and lower limb flexibility, and climbing performance on multiple routes was stated.

Two other tests that were used to assess active static hip flexibility are the Draga- and hip slide test by Draga et al. (170) and Giles et al. (134), respectively. No quality data were reported on either test.

The foot loading flexibility test conducted by Draper et al. (169) and the asymmetry in reach test conducted by Čular et al. (113) combine active static hip flexibility with a climbing movement and are thus more complex compared to tests focused solely on hip flexibility. The inter-session reliability of both tests is rated as high to very high. Čular et al. (113) additionally report an equally high intra-session reliability for the asymmetry in reach test. While they, however, do not report any correlations to climbing ability, Draper et al. (169) report a middle-sized correlation between the results from the foot loading flexibility test and climbing ability.

Two tests were used to assess passive static hip and lower limb flexibility. During the so called froggies, the participants are asked sit or stand with their feet placed together and to then spread their legs as far as possible to the sides. Both studies conducting this test did not provide any data on the test's quality (5, 30). The straddle test, which is also used in other sports, was implemented in three different ways. The implementations differ in the body position of the subjects (lying, sitting, standing) while spreading their legs as far as possible. No data on the reliability of the straddle test are reported. However, a middle-sized negative correlation between the test outcomes in a sitting position and climbing ability was reported by Draga et al. (170). MacKenzie et al. (95) on the other hand report no correlation with climbing ability for males and a low correlation for females.

Wall et al. (131) conducted three different tests to assess frontal hip flexion, hip rotation and leg flexion but did not report any data on test quality.

In summary, fourteen different tests for the assessment of lower limb flexibility in climbing were identified. While high to very high inter-session reliability was reported for six of these tests, mainly low to middle-sized correlations with climbing ability were reported. Only the climbing specific foot raise was reported to highly correlate with climbing ability.

#### 3.2.8. Core strength

The following core strength tests were identified (Table 9):

• Super-man
• Momentum absorption
• Core rotation test
• Body lock off
• Plank
• Sorensen test
• Kraus Weber test battery
• Sit-ups
• Curl-ups
• Fishing kicks
• Leg raise

No quality data are provided for the following tests: core rotation test, plank, Sorensen test, Kraus Weber test battery, sit-ups, and curl-ups. During the fishing kicks tests, participants held on to a bar attached to a 60-degrees overhanging wall. They were then asked to touch a foot plate on the wall with each foot for one second, starting in a vertical position and without swinging their legs. The test was repeated until the plate had not been loaded on three consecutive attempts. Augste et al. (15) reported low to moderate negative correlations to climbing ability. A similar test was conducted by Saeterbakken et al. (86) who report a very high inter-session reliability.

They also report high inter-session reliabilities for the super man and the body lock off test (86). During the super man test, participants adopted a push-up position with their hands on a slide board and their feet against a wall. They were then asked to slide their arms as far forward as possible so they could still return to the starting position. For the body lock off test, participants adopted a horizontal position with one foot on a campus rung and both hands on another. They were then asked to lift their second foot to the same height as the first and to lift their body so that shoulders, pelvis and ankle formed a horizontal line. They then had to hold the position for as long as possible. Augste et al. (15) reported low correlations between “momentum absorption” and climbing ability. For this test participants were asked to position both hands and feet on a 60-degrees overhanging wall. They then simultaneously released both feet and tried to allow as little back swing as possible. Whereas Draper et al. (14) as well as Macdonald and Callender (96) found no significant differences between climbers of different ability levels regarding leg raise measurements, MacKenzie et al. (95) found a low correlation to climbing ability in females and a middle-sized correlation in males.

In summary, eleven different tests were identified to assess core strength in climbing. For six of them no quality data are reported. High reliability measures were reported for body lock off, super-man, and fishing kicks. Low correlations with climbing ability are reported for leg raise and middle-sized to high correlations for “momentum absorption”.

## 4. Discussion

The aim of this review was to give an overview over the quality of different test- and measurement methods for performance, strength, endurance, and flexibility in climbing. The type and frequency of the tests used (Figure 3) correspond to the performance structure of climbing shown in Figure 1. This shows that research is representing the conditional requirements of the climbing sport. Nonetheless, the climbing ability of most samples range across two or more ability levels (IRCRA) and only very few studies focused on specific ability levels. This leads to the fact that only broad assumptions within the field of climbing diagnostics can be made. In addition, all recommendations on testing need to be viewed in context of the population included in the respective study.

Based on current evidence, it is difficult to determine whether individual tests are superior to others in terms of reliability and validity. However, individual tests may be identified as particularly good based on multiple studies and quality checks, while others may need further exploration. Although a large number of studies and tests were included in this review, it should be noted that the majority of the studies (a total of 82 = 55,4%) did not provide data on test quality, which may have biased our analysis.

### 4.1. Performance tests

Climbing and bouldering performance were measured through several tests. Their high complexity and variability are both advantageous and disadvantageous at the same time. On the one hand they can be adapted to focus on various different performance factors such as endurance, strength, climbing ability, dynamics, and kinematics. Additionally, they can be implemented easily and most of them don't require expensive and unwieldy equipment. On the other hand, the fact that they are implemented in various different ways makes it hard to compare the results of different studies. Furthermore, the variability of the routes and walls used lead to substantial differences in the requirements needed to fulfil a test among different ability levels. For example, a test route designed to test climbing endurance in elite climbers might require more strength than endurance in intermediate and advanced climbers.

While there is little quality data reported on performance tests, the correlation between test scores and reported climbing ability is high or up to very high. Especially the repeated ascent of one boulder, and bouldering in a circuit stand out due to a high validity. Even though the test results might seem to be vague, due to the high complexity of the tests, various studies report very high inter-session reliability for top-rope, and treadwall climbing, as well as the rock over climbing test, and the repeated ascent of one boulder. Moreover, studies that evaluated climbing kinematics through expert ratings report high inter-rater reliability. A new attempt to measure climbing performance through climbing kinematics through the assessment of the jerk of the hip trajectory showed high correlations with climbing ability (68, 69). Tests that lack construct validity regarding climbing ability are traverse bouldering, bouldering and lead climbing.

One factor, researchers might criticize about tests that involve bouldering or climbing is the impact of route preview on the test results. While, according to Sanchez et al. (67), route preview does not lead to a climber being more likely to finish the ascent of a route, it is likely to influence the performance on the route itself. The ability to visually inspect a climb before its ascent or not may thus represent a key factor in performance testing (67).

Some climbing performance tests have been used to assess climbing specific endurance. While it was shown that both systemic and localized endurance are important in climbing ability and several tests are needed for a full picture of an athlete (171), there is still no consensus on the most appropriate tests.

In general, five climbing performance tests have not been validated and only eight studies report reliability data. Furthermore, the included population covers different ability levels, which is why no definitive recommendations for climbing performance tests can be given at present.

While we decided to classify the tests according to the exercises performed, another idea would be to classify them according to the intensity of the exercise. To our knowledge, no study has so far distinguished between exhaustive or submaximal tests which would be an interesting topic for future analyses.

### 4.2. Upper limb and finger strength

A total of 16 different test groups for upper limb and finger strength were identified. They were applied by 120 out of 156 studies included in this review. This represents the importance of upper limb and finger maximum strength, muscular endurance and explosive strength in climbing.

All tests conducted to measure finger strength are isometric tests, except for one test by Schweizer and Fuller (102) which is isokinetic. In total, four test groups were identified. However, these consist of almost 230 different ways of implementation regarding hold type, hold depth, arm- and body position, distance between the hands, force thresholds, contraction type, and work to rest ratios. Furthermore, the same tests were modified to assess not only finger isometric maximum strength but also isometric muscular endurance in both sustained and intermittent setups, explosive strength, and maximum strength endurance. The dead hang was reported to have very high reliability ratings by many studies. In addition, acceptable coefficients of variation were reported by Draper et al. (14) and López-Rivera and González-Badillo (82). Only Ozimek et al. (83) report poor CV values (23.4%–29.9%). Both gripping a hand dynamometer and applying force on a hold were also reported to be highly reliable. Acceptable CV-values are additionally reported by multiple studies (4, 96, 115–117, 135). The reliability for pinching a dynamometer has so far only been assessed by in one study (108) reporting very high intra-session reliability. Correlations with climbing ability were on the other hand studied less frequently reported. The dead hang seems to be a valid measure to assess finger isometric muscular endurance and maximum strength. New findings however show that the test is more likely to assess maximum strength rather than muscular endurance (171).

Both gripping and pinching a dynamometer for measuring finger maximum strength seem to be valid ways to assess finger isometric muscular endurance and maximum strength. Applying force on a hold might be a less valid procedure, however all these findings need to be treated with caution as test setups and included populations vary substantially.

One of the tests assessing maximum strength endurance of the fingers that has recently been introduced also assesses finger flexor critical force (132). This parameter is new to climbing research and holds great potential for further investigations of specific strength profiles of climbers and their correlation with climbing ability.

Both gripping a dynamometer and applying force on a hold have been reported to hold high and very high test reliability, respectively, and high levels of standardization in assessing hand strength (172, 173). While we cannot give a final answer to the question which arm- and body positions should be used for finger flexor strength testing, we are able to summarize the current findings in this field. One of the first studies investigating this question found that the most appropriate protocol seems to be to assess maximum grip strength in adolescents with the elbow extended rather than bent at 90 degrees (174). Whether this applies to adult climbers of different ability levels as well, remains to be investigated. Michailov et al. (130), state that, while finger strength testing with arm fixation is more reliable, tests without arm fixation are more related to climbing ability. Amca et al. (175) observed different forms of increase in force with increasing hold depth, depending on the grip technique. This points towards climbers adopting individual choices of body position while climbing according to the chosen grip technique. Consequently, some freedom of choice regarding the type of grip and body positioning during finger strength testing might lead to more reliable and valid results. Baláš et al. (136) assessed the differences between various grip types and report open grip and crimp grip as most closely related to self-reported climbing ability. Additionally, two finger grips might provide more detailed information on individual grip performance variations (136). Bourne et al. (137) assessed the effect of edge depth and found that finger strength measured on deep edges do not predict finger strength on shallow edges. In addition, individual anthropometric factors such as fingertip pulp may influence strength measurements. A recent study by van Bergen et al. (176) suggest to conduct finger strength testing and training with different holds and body positions.

Another factor that many tests differ on is the type of contraction (continuous or intermittent). It was shown that aerobic, alactic, and lactic relative energy contributions differ significantly between both test set ups (138). Researchers and coaches should thus choose the test set up according to the variable they wish to measure. Nonetheless, it remains unclear which work to rest ratio intermittent testing holds the highest correlation to climbing ability in different performance groups. Augste et al. (177) recently published a study aimed at optimizing the correlation of test performance in intermittent finger muscular endurance tests with climbing ability. They found the highest correlations for women and men when 9% and 6% deviation in required force and one second deviation in required pulling time were tolerated, respectively. This might be a good starting point for future research on intermittent finger strength testing.

Low to high reliability and middle-sized correlations to climbing ability have been reported for the assessment of finger flexors RFD. New findings suggest, that RFD plays an important role especially in high elite climbing (178, 179) and should therefore be considered in more detail in future.

As can already be seen form these findings, sex plays an important role in strength testing. Findings by Peterson et al. (180) indicate that relative grip strength measured with a hand dynamometer could be greater in males compared to females due to the decreased hand size of females in relation to males. This has to be taken into account when interpreting forces measured with a hand grip dynamometer.

Two isometric tests assessing upper limb strength were identified. The bent arm hang was used to measure upper limb muscular endurance. When conducted on small holds, however, finger maximum strength also played a role. It was reported to be a reliable test by multiple studies. In addition, diagnostic literature as identified the bent-arm hang as a test with a high level of standardization and a high reliability for young adults (181). Correlations to climbing ability covered a broad range from low to high. Again, the variety of implementations and within sample climbing ability levels is very high. The “best” way to implement this test can thus not be identified. However, it was reported to differentiate between climbers of different ability levels (3, 96, 97). The bent arm hang thus remains a valid test for upper limb strength in climbing. The same was found for the isometric pull up.

Although many dynamic tests to assess upper limb strength in climbing were identified, most of them were applied in only one or two studies (medicine ball throw (31, 112); elbow strength tests (159); biceps strength test (95); shoulder strength test (6, 160); push-ups (31), campus board performance (39, 53); arm jump test (161); bench press (4); pull down (63, 64); traction test (139)). In addition, quality data are only reported for medicine ball throw, the power-slap test and pull-ups. A very high inter-session reliability is reported for all of them by multiple studies. On top of that, Draper et al. (14), Levernier et al. (100), Stien et al. (103) and Laffaye et al. (157) report acceptable CV values for the power slap test and the pull up. While the correlation with climbing ability for these tests only ranges from low to middle-sized, the power-slap test was found to differentiate between different ability levels when assessing upper limb explosive strength and explosive strength endurance (4, 158). Furthermore, the pull up was found to differentiate between boulderers and climbers when assessing upper limb explosive strength (100, 101). In addition, Fetz and Kornexl (172) report a very high level of standardization and high reliability. A high level of standardization and high inter-rater reliability are also reported for the medicine ball throw when performed in a standing position by Bös and Schlenker (181). While no quality data was reported for push-ups, Bös and Schlenker (181), and Fetz and Kornexl (172) state a high level of standardization and high inter-session reliability for push-ups performed with a clap behind the back after every repetition. Augustsson et al. (159) were the only ones to report data on elbow strength. While this test seems to be a valid test especially in bouldering, further analysis need to be conducted.

This shows that even though climbing is often characterized as a series of isometric contractions, and dynamic tests are not often used, dynamic explosive strength of the shoulders and upper arms plays an important role in climbing and should thus be included into performance assessments in addition to isometric tests.

### 4.3. Upper limb endurance

Although upper limb endurance is an important factor in climbing, it was only investigated by a total of seven of the 156 included studies. Reliability measures for rowing ergometry are only reported by one study, while two report correlations to climbing ability. High correlations are reported for maximum strength assessed through the one repetition maximum and endurance assessed through maximum oxygen consumption. While no data on the reliability of arm crank ergometry are reported by the included studies, the test has been shown to hold a high inter-observer and inter-session reliability by Bulthuis et al. (182). However, only low to middle-sized correlations with climbing performance are reported by one study for arm crank ergometry.

These findings suggest that both tests could be valid for the assessment of upper limb endurance in climbing. However, more research by multiple studies is needed in this field (178).

### 4.4. Upper limb flexibility

While upper limb flexibility is reported to be one of the key factors of climbing (6), only two studies have assessed active dynamic shoulder flexibility. Additionally, only one study reports data regarding test quality (6). General diagnostic literature has already shown that shoulder flexibility assessed with a scaled rod moved over the head with straight arms is a measure with very high objectivity, and high intra-session reliability (183). However, more research regarding upper limb flexibility in climbing is needed to be able to provide test recommendations.

### 4.5. Lower limb strength

Lower limb strength was reported to be a key factor in climbing. In addition, coaches report an increasing importance of lower limb strength in modern bouldering and speed climbing (184). Nonetheless, very few studies included lower limb strength tests into their test batteries. The studies that did include lower limb strength tests mainly focus on lower limb explosive strength. Only one of the seven tests found focuses on maximum strength and one on lower limb muscular endurance. This is in line with the results of Mermier et al. (6) who found that lower body explosive strength plays an important role in climbing ability.

Nonetheless, hardly any data is reported on test quality. It can only be assumed that the jump with high foot (15), has high to very high inter-session reliability. The authors, however, emphasize that this test should only be included in a test battery if both angular position of the knee and test performance are closely monitored (15). All tests for which correlation values to climbing ability are reported, show low to high correlation with climbing ability. Nevertheless, this information should be taken with caution, as it is based only on the results of single studies and is therefore not conclusive. As shown by Krawczyk et al. (79) lower limb strength is a key factor, in speed climbing, and this relationship should thus be evaluated further. General sports diagnostics have shown that the standing long jump shows a very high level of standardization, and middle-sized to high inter-session reliability (172). In addition, both vertical jump and one legged squats have been shown to hold a high inter-session reliability in general strength testing (172, 181, 185) which is a good starting point for future climbing-specific assessments to provide valid test recommendations.

### 4.6. Lower limb endurance

Lower limb endurance was not reported to be a key factor of climbing. Nevertheless, six studies included treadmill running or cycle ergometry into their test batteries. The aim of the studies was to compare the respiratory requirements of running or cycling with those of climbing. While only three of the studies report low correlations with climbing ability, all indicate that climbing ability is not dependent on aerobic capacity as determined by a traditional treadmill analysis or cycle ergometry (37, 41, 42, 45, 95). In addition, no study reports reliability data, which shows another gap in climbing research. Nonetheless, it has been shown that incremental treadmill tests are a reliable tool for measuring lactate thresholds, blood lactate concentrations, and maximum oxygen consumption (186). It can be concluded that traditional lower limb endurance tests most probably do not directly contribute to climbing ability and should thus not be included in performance analysis.

### 4.7. Lower limb flexibility

As supported by multiple studies, lower limb flexibility is a key performance component of climbing (3, 6). However, the test battery included lower limb flexibility tests in only a few studies. For all tests for which inter-session or intra-session reliability data are reported, the reliability is very high. Additionally, Čular et al. (113) report an acceptable CV and SEM for the right and left hand individually, but not for the absolute values in the asymmetry in reach test. The high reliability of the flexibility tests is in line with diagnostic literature reporting high inter-rater and inter-session reliability for the sit and reach and the straddle test (172, 183). In contrast, the correlation to climbing ability ranges from middle-sized to high only for the climbing specific foot raise and the foot loading flexibility test and is low for the remaining tests. While researchers have emphasized that climbing specific flexibility tests are superior to less specific tests (169), our results show that both specific tests performed on a climbaflex board and existing tests used in many other sports only show low to middle-sized correlations with climbing ability. This could indicate that despite previous findings lower limb flexibility is a less important factor in climbing. Another possible explanation could be that due to their complexity these tests might not only refer to flexibility. The asymmetry in reach test for example might also include factors of shoulder strength. In addition, the current state of research may not be strong enough to support either position. As the samples of most studies focusing on lower limb flexibility range from lower level to elite or even to higher elite climbers, no ability group has specifically and thoroughly been investigated until now. More research in this area is thus needed and should thus focus on specific ability groups.

### 4.8. Core strength

Even though core strength was reported to be a key component of climbing, only 11 out of 156 studies conducted core maximum strength tests and muscular endurance tests of the core. Diagnostic literature reports high intra- and inter-tester, as well as high inter-session reliability for the Sorensen test, sit-ups, curl-ups, and leg raise (181, 185, 187). In climbing specific research, however, only one study reports reliability data and only two report on the validity of a single test each. While the inter-session reliability of the super-man, the body-lock, and fishing kicks are reported to range from high to very high, the correlations reported for the leg raise and “momentum absorption” range from low to high only. This again highlights the need for further research in the field of strength testing in climbing.

### 4.9. Practical applications

The large variety of tests used, and the large number of factors influencing the measured values (ability level, wall inclination, loads, test implementation, etc.), makes it hard to give concrete test recommendations to coaches and researchers. Our suggestions reflect the current state of evidence; we only recommend tests with high validity.

According to our findings, the most valid tests for bouldering endurance, climbing performance, and climbing kinematics are the repeated ascent of one boulder, lead climbing, and top-rope climbing, respectively. Finger maximum strength is best assessed through applying force on a hold, rather than using a hand dynamometer. Intermittent dead hang protocols are reliable and valid tests for finger muscular endurance. Upper limb maximum strength and strength can be measured through the bent arm hang and pull-ups. Isometric pull-ups additionally allow the assessment of explosive strength, for which the power-slap test can also be used. Regarding the lower limbs, currently no test can be recommended due to low or missing validity.

## 5. Conclusion

When creating a test battery and comparing and analyzing test results, researchers are almost overwhelmed by the multitude and variability of diagnostic options. To date, no between test correlation analysis or multiple regression analysis has been carried out to find out whether it might be sufficient to perform only few tests in order to successfully map climbing ability. Of course, this does not apply to diagnostics which aim to identify deficiencies or weaknesses. However, when evaluating training effects, for example, a reduced test battery could save a lot of time and work.

While some tests have been validated mainly in the area of upper limb and finger strength, especially the assessment of climbing performance, core strength, global endurance, and lower limb strength and flexibility lack valid and reliable testing methods. Standardized settings such as the moon or the kilter board have not been used to assess performance to this day and might hold potential for future examinations within performance testing.

This review might give the impression that in order to reach a “perfect test”, authors should strive towards optimized reliability and validity measures. While low-complexity tests are not characterized by a particular proximity to climbing, they might, however, lead to significantly more reliable test results. This is why the aim of this review was not to find the test with the highest quality data reported. Instead, it was our aim to give an overview of the variety of tests and their current state of quality assessment. Researchers can use this information to create future test batteries or to further assess test quality.

In this context it also has to be kept in mind that the term “climbing specific” is not clearly defined to this day due to the great complexity and variability of the climbing movement. As already postulated by Stien et al. (188), further biomechanical analyses of the climbing movement need to be conducted to formulate concrete test recommendations. During the last years, for example, coaches have reported an increasing importance of lower limb coordination (184) in bouldering and speed climbing.

On top of that, we were able to confirm that discipline-specific tests do not exist in climbing to this date. Many studies did not include the discipline, the climbing ability, reported by the participants, was related to. This makes it hard to give coaches discipline-specific advice which is why we ask authors to specifically name the climbing discipline used to calculate correlations with the test results in future. Nonetheless, it has to be taken into account, that our goal was to conduct a generic review regarding diagnostics in climbing which is why our literature search might not have allowed us to identify some discipline-specific studies. Future research could focus on this topic.

As criticized by Stien et al. (188) and confirmed in this review, research on testing in climbing lacks data on test quality. Future research on strength, endurance and flexibility in climbers should thus aim to provide detailed information on the test reliability and validity. Furthermore, authors should strive to use similar tests in future studies to increase comparability of test results. First steps towards a uniform test battery have already been taken recently (14) and should be followed up in future as they are not only important for research. Test results should also form the basis for training organization (189) and are a key factor of injury prevention (184).

Furthermore, inadequate descriptions regarding the ability level, sex and main discipline of the subjects examined in the studies also posed a major challenge in the context of this review. The IRCRA scale (18), introduced a few years ago, has enabled a uniform assessment of performance. In addition, future research should include clear information on the subject's sex and main discipline.
