# Supplementary material for: Physical performance testing in climbing—A systematic review
Source: Front Sports Act Living. 2023 May 9;5:1130812. doi: 10.3389/fspor.2023.1130812 (PMC10203485; doi:10.3389/fspor.2023.1130812)
Supplement: Supplementary file 1 [file Table1.pdf]

Supplementary Material

List of abbreviations

*	significant (p<.05)
**	significant (p<.01)
***	significant (p<.001)
adv.	advanced climbers
b	boulderers
c	climbers
CCC	concordance correlation coefficient
CF	critical force
$CI_{95\%}$	confidence interval 95%
corr.	correlation
CV	coefficient of variation
d	days
diff.	difference/differences
E	endurance
EMG	electromyography
ESE	explosive strength endurance
ES	explosive strength
ER	external rotation
f	female
FCR	flexor carpi radialis
FDP	flexor digitorum profundus
FLEX	flexibility
fR	respiratory rate
HR	heart rate
ICC	intraclass correlation coefficient
inter.	intermediate climbers
IR	internal rotation
LoA	limits of agreement
m	male
MD	mean difference
MMG	mechanomyography
MS	maximum strength
MSE	maximum strength endurance
MVC	maximum voluntary contraction
nd	no data regarding the correlation
ns	non-significant
nv	no values regarding the significance
RCP	respiratory compensation points
RER	respiratory exchange ratio
RFD	rate of force development
sc	speed climbers
ME	muscular endurance
VCO_2	carbon dioxide production
VE	minute ventilation
VO_2	oxygen consumption
V_T	tidal volume
x/x/x	shoulder flexion (°) / elbow flexion (°) / shoulder adduction or abduction (°)
–	no data reported

Supplementary Table 1. Implementation variations, objects of measurement, measured values and units, data on quality criteria and further information on the repeated ascent of one boulder.

**repeated ascent of one boulder**	
**Object of measurement:**<br>measured value (unit)	**bouldering endurance**<br>time to failure (s)
**Reliability**	*inter-session*<br>*2d* **elite m**: r=0.99*, ns diff. betw. trails (34)
**Validity**	**elite m:** linear corr. with intermit. finger hang time, r=0.87*** (34)
**Additional data reported:** training effects (35)	

Supplementary Table 2. Implementation variations, objects of measurement, measured values and units, data on quality criteria and further information on bouldering in a circuit.

**boulder in a circuit**<br>continuous (incremental) bouldering	
**Object of measurement:**<br>measured value (unit)	**bouldering endurance**<br>time to failure (s)[1]<br>VE (L/min)[2]<br>VO_2, VCO_2 (ml/min/kg)[3]<br>HR (bpm)[4]<br>RER (#)[5]<br>number of moves completed (#)[6]<br>wall angle at point of failure (°)[7]
**Validity**	**lower grade-elite m:** r=0.89, R^2=0.79 (ns)[7]; r=-0.82 at 90°*[3], r=-0.84 at 105°*[3]; r=-0.43 at 90°*[4], r=-0.78 at 105°*[4], higher level c tended to achieve higher VE/ VO_2 ratio (hyperventilation) than lower level c and attained higher RER (ns)[3, 5] (37)
**Additional data reported:** prefatigued effect (36)[6]<br>**No data on quality criteria reported:** (26)[-]	

Supplementary Table 3. Implementation variations, objects of measurement, measured values and units, data on quality criteria and further information on bouldering in a circuit.

**boulder traverse**<br>hard | easy<br>half crimp | pinch | inclined holds | horizontal holds	
**Object of measurement:**<br>measured value (unit)	**bouldering endurance**<br>time to failure (s)[1]<br>number of moves completed (#)[2]<br>RER (#)[3]<br>HR (bpm)[4]<br>VO_2 (mo/min/kg)[5]<br>lactate$_{pre, 3, 10, 20 min post}$ (mmol/L)[6]
**Validity**	**elite-higher elite m:** higher on easy than on hard traverse**[1, 3]; r=0.7*[6]; hard traverse: r=0.8-0.94*[1], easy traverse: r=0.52-0.60*[1], corr. betw. VO_2 and duration of hard and easy traverse r=0.8-0.85* (38)
**Additional data reported:** supplementation effects (39)[2], hold type effects (38)[1, 3-6]	

Supplementary Table 4. Implementation variations, objects of measurement, measured values and units, data on quality criteria and further information on treadwall climbing.

<table>
<tr><td colspan="2">**treadwall climbing**
continuous | discontinuous
consistent | incremental (increasing speed/increasing wall inclination)</td></tr>
<tr><td>**Object of measurement:**
measured value (unit)</td><td>**climbing endurance**
HR (bpm)[1]
VO2 (L/min)[2]
RER (#)[3]
V_T (L)[4]
VE (L/min)[5]
fR (brpm)[6]
FDP oxygen saturation (%)[7]
gas exchange threshold (s)[8]
muscle oxygenation breakpoint (s)[9]
$lactate_{0, 1, 3 \text{ min post}}$ (mmol/L)[10]
number of moves (#)[11]
time to failure (s)[12]
peak angle (°)[13]</td></tr>
<tr><td>**Reliability**</td><td>*inter-session (2d)*
adv.-elite m: r=0.99** (38)[7, 10, 12]</td></tr>
<tr><td>**Validity**</td><td>**adv.-higher elite>inter.-elite m/f** *** (47)[12]
inter. m: associated with highest self-reported RP grade**(45)[2]
elite vs. adv. m: diff. in regression line slope**, regression line equations for **elite** (R^2=0.83*) and **adv.** (R^2=0.58*) paralleled each other and diff. sig. only for the intercept (nv) (42)[1, 2]
elite>inter. m: d=1.46***[13], d=0.95*[4], d=1.48***[12]; **inter.>elite.** d=0.25-0.55 (ns)[6]; **inter.-elite** R^2=0.70 (nv)[13], R^2=0.66 (nv)[10]; **inter.~elite** d=0.02 (ns)[5], d=0.04 (ns)[3]; corr. with impulse from intermittent hangboard test R^2=0.71 (nv) but not with max. strength R^2=0.06 (nv)[9] (41)
adv. m/f: r=0.59-0.66 (43)[13]</td></tr>
<tr><td colspan="2">**Additional data reported:** training effects (46)[1, 12], (40)[1, 10, 11]
No data on quality criteria reported: (44)[1, 2, 10]</td></tr>
</table>

Supplementary Table 5. Implementation variations, objects of measurement, measured values and units, data on quality criteria and further information on top-rope and lead climbing.

<table>
<tr><td colspan="2">**top-rope and lead climbing**
lead one route then top-rope same or different route</td></tr>
<tr><td>**Object of measurement:**
measured value (unit)</td><td>**climbing kinematics**
HR (bpm)[1]
score on observer scale (#)[2]</td></tr>
<tr><td colspan="2">**Additional data reported:** anxiety effects (49)[1, 2]
No data on quality criteria reported: (28)[2]</td></tr>
</table>

Supplementary Table 6. Implementation variations, objects of measurement, measured values and units, data on quality criteria and further information on outdoor climbing.

<table>
<tr><td colspan="2">**outdoor climbing**</td></tr>
<tr><td>**Object of measurement:**
measured value (unit)</td><td>**climbing endurance**
VO_2, VCO_2 (ml/min/kg)[1]
VE (%)[2]
$lactate_{3, 5, 8 \text{ min post}}$ mmol/L)[3]
climbing time (s)[4]</td></tr>
<tr><td>**Validity**</td><td>**elite<lower grade m*** (48)[3, 4]</td></tr>
</table>

Supplementary Table 7. Implementation variations, objects of measurement, measured values and units, data on quality criteria and further information on the rock over climbing test.

<table>
<tr><td colspan="2">**rock over climbing test**</td></tr>
<tr><td>**Object of measurement:**
measured value (unit)</td><td>**climbing ability**
maximum height held for 2 s (cm)</td></tr>
<tr><td>**Reliability**</td><td>*inter-session (7-14d)*
lower grade-higher elite m/f: ICC=0.90 (50)</td></tr>
<tr><td>**Validity**</td><td>**m/f: lower grade < elite****, **adv.** and inter.****,
inter.<elite** and **adv****, **adv.>lower grade****,
and **inter.****, **inter.>lower grade**** (50)</td></tr>
</table>

Supplementary Table 8. Implementation variations, objects of measurement, measured values and units, data on quality criteria and further information on bouldering.

**bouldering**<br>2-3 problems (in a competition)<br>6:6 min | 5:5 min | 4:3 min | 4 min:– | –<br>progressing level | –	
**Object of measurement:**<br>measured value (unit)	**bouldering level**<br>number of attempts (#)[1]<br>number of attempts per top and zone (#)[2]<br>number of grips (s)[3]<br>attempt time (s)[4]<br>recovery time (s)[5]<br>climbing time (s)[6]<br>viewing time (s)[7]<br>hand contact time (s)[8]<br>reach time (s)[9]<br>dynamic[10] and static time (s/s)[11]<br>number of moves completed on best attempt (#)[12]
**Reliability**	*intra-session*<br>**no data m:** CV=0.7% ($CI_{95\%}$=0.4-1.9)[4]; CV=1.1% ($CI_{95\%}$=0.7-2.7)[5]; CV=0.9% ($CI_{95\%}$=0.6-2.3)[6]; CV=0.9% ($CI_{95\%}$=0.6-2.3)[7]; CV=2.0% ($CI_{95\%}$=1.3-5.0)[8]; CV=21.2% ($CI_{95\%}$=14.1-68.1)[9]; CV=3.2% ($CI_{95\%}$=1.9-8.2)[10]; CV=3.3% ($CI_{95\%}$=2.0-9.9)[11] (51)
**Validity**	**no data m:** corr. with group ranking and world championships ranking respectively r=0.01-0.03 (ns)[7]; r=0.36-0.50*[2]; r=-0.44- -0.47*[1]; r=0.10-0.12*[3]; r=0.35-0.39*[5]; r=-0.36- -0.39*[4]; r=0.29-0.33*[6] (52)<br>**no data:** pos. link with bouldering experience and results on most difficult problem, greater group mean on most difficult problem*, ns greater scores on other problems for subjects who had competed before (55)[12]
**Additional data reported:** training effects (7)[1], (53)[1]<br>**No data on quality criteria reported:** (54)	

Supplementary Table 9. Implementation variations, objects of measurement, measured values and units, data on quality criteria and further information on top-rope climbing.

**top-rope climbing**<br>one route at different angles | attempt challenging route (success – next harder one; failure – easier one) | one route multiple times until failure | one progressively harder route until failure |1-3 routes with/without preview, normal pace or as fast as possible			
**Object of measurement:** measured value (unit)	**climbing endurance/level/speed**<br>VO_2, VCO_2 (ml/kg/min)[1]<br>RER (#)[2]<br>HR (bpm)[3]<br>highest hold reached (on hardest route attempted) (#)[4]<br>time to failure (s)[5]<br>climbing time per route (s)[6]<br>number of arm movements (#)[7]<br>lactate10 min post (mmol/L)[8]<br>climbed distance (m)[9]<br>oxygen cost (ml)[10]	**climbing kinematics**<br>number and duration of stops (#)[11]<br>exploratory and performative movements (#)[12]<br>score on observer scale (#)[13]<br>jerk of hip trajectory and hip orientation (#)[14]<br>geometric entropy of the hips (#)[15]	**climbing dynamics**<br>vertical rection force under foot (N/kg·s)[16]
**Reliability**	*inter-session (2d)*<br>**lower grade m/f:** $ICC_{(2, 1)}$=0.97 (nv) (59)[4]<br>**inter.-adv. m/f:** diff. betw. trials d=0.69*[7], r=0.10 (ns)[5], r=0.48 (ns)[8] (62)	*inter-session (2d)*<br>**inter. no data:** diff. betw. sessions ($F_{(1.05,7.348)}$=5.18*, η_P^2=0.428) (68)[14]<br>*intra-session*<br>**inter. no data:** trial-to-trial changes according to practice route (69)[15]<br>inter-rater<br>**inter. f:** r=0.88*(70)[13]	-
**Validity**	**elite>inter.-adv. no data***[7]; **elite<inter.-adv.***[10]; **elite vs. inter.-adv.:** sig. diff.*[2-3, 8], ns diff.[1] (75)<br>**inter. m/f:** ns diff. betw. m and f (24)[6]	**inter. no data:** corr. betw. normalized jerk of hip trajectory and normalized jerk of hip orientation r=0.99*** (68)	**f: lower grade** lower vertical loading on foot holds and higher physiological responses than **inter.-adv.** corresponding to lower HR and RER; ns age effect (71)[1-3, 16]
**Additional data reported:** training effects (56, 57, 59, 60)[4], (62)[5, 9], (70)[13]; recovery effects (61)[3,5, 7, 8], (65)[9]; preview effects (67)[11, 12]; route effects (68)[14, 15], (69)[15], (71)[16]<br>**No data on quality criteria reported:** (58, 63, 64)			

Supplementary Table 10. Implementation variations, objects of measurement, measured values and units, data on quality criteria and further information on lead climbing.

<table>
<tr><td colspan="4">**lead climbing**
1-2 routes OS | ascend and descend 5 routes | (simulated) competition</td></tr>
<tr><td>**Object of measurement:** measured value (unit)</td><td>**climbing endurance/level**
climbing time (s)[1]
number of moves completed/ highest hold reached (#) or (%)[2]
HR (bpm)[3]
$lactate_{post}$ (mmol/L)[4]
mean difficulty (#)[5]</td><td>**climbing kinematics**
score on observer scale (#)[6]
climbing pace (m/s)[7]
geometric entropy (#)[8]
contact time (s)[9]</td><td>**climbing dynamics**
impulse (Ns)[10]
ratio tangential to normal force (#)[11]
smoothness factor (#)[12]
maximal and force (N)[13]
hausdorff dimension (#)[14]</td></tr>
<tr><td>**Reliability**</td><td>*inter-session (7-14d)*
lower grade-elite m/f: corr. betw. two climbing routes r=0.81 (nv) (measured similar climbing abilities) (6, 47)[2]</td><td>*inter-session (4wk)*
inter.-adv. m/f: total scores r=0.92 (nv), sub-scores (upper and lower part of the wall) r=0.71-0.91 (nv) (2, 74)[6]

inter-rater
inter.-adv. m/f: reliability betw. observer scores r>0.81 ($CI_{95\%}$=0.61-0.80) (2, 74)[6]</td><td>-</td></tr>
<tr><td>**Validity**</td><td>**lower grade-elite m/f:** r^2=0.59*** (6, 3)[2]
lower grade-higher elite m: r=0.45*; lactate recovery indicator ξ r=0.69***(3, 73)[4]
elite m: correlation with route rating ρ=-0.75, df=17*** (3, 76)[2]</td><td>**m/f: inter.-adv.** sig. diff. betw. successful and unsuccessful c**[7-8]; **inter.-adv.** >50% variation in OS grades explained by scores on observer scale, geometric entropy only explained small percentages of variation and climbing pace explained 52% (2, 74)[6, 8]</td><td>**inter. m/f:** hausdorff dimension proved to represent and replace all other mechanical parameters (2, 77)[10-14]</td></tr>
<tr><td colspan="4">**Additional data reported:** training effects (72)[1-4], (7)[2]; anxiety effects (49)[6]
No data on quality criteria reported: (75)[-]</td></tr>
</table>

Supplementary Table 11. Implementation variations, objects of measurement, measured values and units, data on quality criteria and further information on one speed climbing run.

<table>
<tr><td colspan="2">**1 speed climbing run**</td></tr>
<tr><td>**Object of measurement:**
measured value (unit)</td><td>**climbing ability**
climbing time (s)</td></tr>
<tr><td colspan="2">**Additional data reported:** training effects (33)
No data on quality criteria reported: (79)</td></tr>
</table>

Supplementary Table 12. Implementation variations, objects of measurement, measured values and units, data on quality criteria and further information on the speed climbing start.

<table>
<tr><td colspan="2">**speed climbing start**</td></tr>
<tr><td>**Object of measurement:** measured value (unit)</td><td>**climbing dynamics**
impulse (N)[1]
direction of impulse (#)[2]</td></tr>
<tr><td>**Validity**</td><td>**elite-higher elite no data:** linear corr. with experience, diff. foot positions changed direction of impulse by up to 30° in a plane parallel to wall (nd) (78)[2]</td></tr>
</table>

Supplementary Table 13. Implementation variations, objects of measurement, measured values and units, data on quality criteria and further information on the dead hang.

**dead hang:**<br>open crimp | half crimp | slope grip | pinch grip | self-chosen grip | metal bar | ice axis | –<br>6 | 8 | 10 | 11 | 12 | 14 | 15 | 19 | 20 | 23 | 25 | 30 | 40 | – mm<br>shoulder width | self-chosen grip width | –				
**Object of measurement:** measured value (unit)	**finger isometric ME:**<br>time to failure (s) [1]<br>minimum edge depth 40s-hang (mm) [2]<br>lactate (mmol/L) [3]<br>blood pressure (mmHg) [4]<br>muscle oxygen saturation (%)[5]	**finger isometric intermittent ME:**<br>number of repetitions (#)[6]	**finger isometric MS:**<br>weight held for 3, 5 or 7s (kg)[7]	**finger isometric ME /MS:**<br>dead hang on small edges time to failure (s)[8]<br>one-arm dead hang time to failure (s)[9]
**Reliability**	*inter-session*<br>*7d* **adv.-elite m/f:** ICC=0.89 ($CI_{95\%}$=0.60-0.97)[1], ICC=1.00 ($CI_{95\%}$=1.00-1.00)[2] (81)<br>*7d* **elite-higher elite m/f:** ICC=0.91 ($CI_{95\%}$=0.41-0.99)[1], ICC=0.99 ($CI_{95\%}$=0.89-1.00)[2] (81)<br>*1d* **lower grade-elite m/f:** ICC=0.88 ($CI_{95\%}$=0.835-0.915), inacceptable variation for lower grade f (>30%) (14)[1]<br>*7d* **elite no data:** ICC=0.86 ($CI_{96\%}$=0.56-0.96) - one measurement, ICC=0.92 ($CI_{96\%=}$0.72-0.98) – mean; CV=12.8% (82)[1]<br>*7d* **elite m**: ICC=0.13-0.73, **adv.-elite:** CV=23.4-29.9% (83)[1]	*inter-session (2d)*<br>**inter.-elite m:** ns diff., **elite** ICC=0.97 ($CI_{95\%}$=0.36-0.96) (78)[6]<br>**elite m:** ns diff.; r=0.86* (34) [6]	*inter-session (7d)*<br>**adv.-elite m/f:** ICC=0.99 **(**$CI_{95\%}$=0.89-1.00) (2)[7]<br>*7d* **elite-higher elite m/f:** ICC=0.99 ($CI_{95\%}$=0.98-1.00) (81)[7]<br>**adv.- elite m:** CV=22.0-29.9% (83)[7]<br>**elite no data** ICC=0.93 ($CI_{95\%}$ =0.77-0.98) - one measurement, ICC=0.96 ($CI_{95\%}$=0.87-0.99) – mean; CV=7.8% (82)[7]	-
**Validity**	**adv.-elite m/f:** r=0.62** ($CI_{95\%}$=0.72-0.83) (81)[1]<br>**elite-higher elite m/f**: r=0.77*** ($CI_{95\%}$=0.47-0.91) (81) [1]<br>**lower grade-elite m/f:** performed well in differentiating (14)[1]<br>**lower grade-higher elite m:** r=0.87 (nv), **f:** r=0.87 (nv) (84) [1]<br>**inter.-adv. m/f:** r=-0.26 (ns) (62)[1]<br>**no data m/f:** ns age group differences (54)[1]<br>**elite.-higher elite. m:** R=0.52* (87)[1]<br>**adv.-elite m:** r=0.53*-0.57 (ns) (83)[1]<br>**lower grade-elite m:** r=0.83* (85)[1]<br>**elite m/f:** best c performed best in initial test, r=0.62*** (91)[1]<br>**adv.-elite m/f:** r=-0.57** ($CI_{95\%}$= -0.80- -0.20) (81)[2]<br>**elite-higher elite m/f**: r=-0.73*** ($CI_{95\%}$= -0.89- -0.40) (81)[2]<br>**no data m:** r=0.87* (27)[3]	**no data m/f:** proved to be empirically and statistically relevant to performance in at least one of the competition disciplines of sport climbing (32)[6]	**adv.-elite m/f:** r=0.58** ($CI_{95\%}$=0.21-0.80) (81)[7]<br>**elite-higher elite m/f:** r=0.84*** ($CI_{95\%}$=0.61-0.94)[7]<br>**differentiates higher elite m:** CV=25,39% (87)[7]<br>**adv.-elite m:** r=0.7*** (83)[7]<br>**elite m/f:** best c best in initial test (91)[7]	**no data m/f:** proved to be empirically and statistically relevant to performance in at least one of the competition disciplines of sport climbing (32)[9]
**Additional data reported:** training effects (86, 89)[1], (90)[1, 5], (34, 103)[6], (82, 88, 91)[7], (33)[9]; rest condition effects (86)[1], grip type effects (80)[1]<br>**No data on quality criteria reported:** (63, 64, 92)[5], (15)[6], (93)[7], (25)[8], (62)[-], (31)[-]				

Supplementary Table 14. Implementation variations, objects of measurement, measured values and units, data on quality criteria and further information on the bent arm hang.

**bent arm hang:**<br>open crimp | half crimp | jug | metal bar | –<br>25 | 40 | 45 | – mm<br>shoulder width | biacromial width + 2 fists | –	
**Object of measurement:** measured value (unit)	**upper limb + finger isometric ME/MS:**<br>time to failure (s) [1]<br>one arm bent arm hang time to failure (s)[2]<br>one arm bent arm hang max. weight held for 3 s (%)[3]
**Reliability**	*inter-session*<br>*2d* **lower grade-elite m/f:** ICC=0.89 ($CI_{95\%}$=0.85-0.93) (81)[1]<br>*7d* **adv.-elite m/f:** r=0.97-0.99 (nv) (15)[3]
**Validity**	**inter-elite: m:** r=0.23 (ns), **f:** r=0.3 (ns) (94)[1]<br>**lower grade-elite m/f:** performed well in differentiating performance levels (14)[1]<br>**lower grade-higher elite: m:** r=0.70 (nv), **f:** r=0.80 (nv) (84)[1]<br>**nc and lower grade-adv. m:** ns diff. (98)[1]<br>**nc<inter. f*** (97)[1]<br>**elite>inter**.* and **nc m*** (3)[1]<br>**elite>nc m***** (96)[1]<br>**lower grade-adv. m:** r=0.71***; **f:** r=0.72*** (95)[1]<br>**inter.-higher elite m**: r=0.69*** (99)[1]<br>**no data m/f:** older>younger participants**[1], ns age group differences [2] (2, 54)<br>**lower grade-adv. m/f:** r^2=0.59*** (2, 6)[1]<br>**no data no data m/f::** proved to be empirically and statistically relevant to performance in at least one of the competition disciplines of sport climbing (2)[1]<br>**lower grade-adv. m/f:** lacked differentiation (14)[2]<br>**adv.-elite m/f: b** r>0.8 (nv), **c:** r=0.65 (nv), **sc:** r>0.7 (nv) (15)[3]
**Additional data reported:** training effects (46)[1]<br>**No data on quality criteria reported:** (30, 63, 64)[1], (31)[-]<br>**Diagnostics literature:** reliability: high level of standardization; r=0.82-0.91 (nv) for participants aged 11-19 yrs.; validity: no correlation betw. hangtime and half time of 70% MVC MS in people aged 11-50 (181)	

Supplementary Table 15. Implementation variations, objects of measurement, measured values and units, data on quality criteria and further information on the pull-up.

**pull-up**<br>normal[1]/explosive[2]/one handed horizontal[3]/two finger[4]/added weight[5]/isometric[6]<br>50cm apart/shoulder width/1.5 x biacromial breadth/ no data<br>jug/rung (10, 14, 18, 22, 23, 30, 80 mm)/bar (25, 40 mm)/ no data<br>half crimp/ open crimp/jug/ pinch/ open grip/ preferred grip/ no data						
**Object of measurement:** measured value (unit)	**upper limb concentric ES[2]**<br>power$_{highest\ mean\ and\ peak}$ (W)[a]<br>velocity$_{highest\ mean\ and\ peak}$ (m/s)[b]<br>force production (N)[c]	**upper limb ESE[1]**<br>number of repetitions in 20 s (#)[d]	**upper limb concentric-eccentric ME[1]**<br>number of repetitions (#)[e]<br>maximum power (W)[f]<br>maximum force (N)[g]<br>EMG (mV)[h]	**upper limb + finger concentric-eccentric MSE[3, 4]**<br>- [i]	**upper limb + finger isometric MS[5]**<br>1RM (kg)[j]	**upper limb isometric ES and upper limb + finger MS[6]**<br>RFD $_{50, 100, 150, 200, 250\ ms\ /\ start\ to\ peak}$ (N/s)[k]<br>time to reach 25, 50, 75, 95, 100% of maximum strength (s)[l]<br>peak force (N)[m]<br>average force (N)[n]
**Reliability**	*inter-session (same day)*<br>**adv.-higher elite m:** ICC=0.84-0.95 (CV=2.75%-6.62%)[2b]; ICC=0.98-0.99 (CV=1.00%-6.18%)[2c] (100)	-	*inter-session*<br>*1d* **lower grade-elite.:** ICC=0.97 ($CI_{95\%=}$0.92-0.99), inacceptable variation in lower grade f (>30%) (14)[1e]<br>*7d* **adv.-elite m:** CV=21.04-21.68 (83)[1e]<br>*intra-session*<br>**inter.-adv. m:** R=0.96-0.99 (nv) (102)[1e]	-	*inter-session*<br>*7d* **adv.-elite m:** CV=7.7 (83)[5j]	*intra-session*<br>*same day* **inter.-elite m:** ICC:0.88-0.99, **inter.-adv.:** CV=16.9-31.3%, **elite:** CV≤10% for $RFD_{100\ and\ 250\ ms\ from\ onset}$ (53)[6k]<br>*5 wks* **adv.-elite m:** $RFD_{absolute}$: CV=8.11%, RFD_{100}: CV=0.83%, $F_{max-avg\ (1500\ ms)}$: CV=4.72% (53)[6k]
**Validity**	**inter-adv. m/f:** b>c* (101)[2b]<br>**adv.-higher elite m:** sig. diff. betw. b and c for $power_{max}$ **, $slope_{force-velocity}$*, $velocity_{max}$*, power* and betw. **b** and **sc.** for P_{max}*, $velocity_{max}$**, and power** (100)	-	**inter.>lower grade. m; nc-inter.:** ns diff. (3)[1e]<br>**lower grade-elite. m/f :** strong construct validity (no differentiation betw. inter. and adv.) (nv) (14)[1e]<br>**nc-inter. f:** ns diff. (97)[1e]<br>**inter.-adv.: m:** r=0.71***; **f:** r=0.72*** (ns) (95)[1e]; **m:** r=0.08 (ns); **f:** r=0.08 (ns) (94)[1e]<br>**no data m/f:** older>younger** (54)[1e]<br>**adv.-elite m:** ns diff. (87)[1e]; r=0.53- 0.57* (83)[1e]	-	**elite m:** **>beginners:** from study (nv) (104), (30)[5j]<br>**adv.-elite m:** ns diff. (87)[5j]	**inter.-adv. m:** ns diff., **elite>inter. and adv.****, **elite>inter.** at RFD_{100}* and RFD_{150}*, **>adv.** at RFD_{50}* and RFD_{100}* (103)[6k]<br>**inter.-elite. m:** ns diff. (103)[6l]<br>**elite>inter. m***** and **adv.*****, ns diff. betw. **inter.** and **adv.** (103)[6m]<br>**inter.-elite m:** r=0.77***[6m], r=0.61***[6k], r=0.73*[6n] (85)<br>**inter-adv. m:** ns corr. to performance (85)[6m]<br>**inter.-adv. m/f: b>c*** (101)[6k,n,m]
**Additional data reported:** blood flow restriction effects (105)[2a-b]; hold type effect (106)[1e-h], (101)[6k, n, m], (102)[1e] ; supplementation effects (46)[1e]; (102)[1e], training effects (189)[6k, l, m], (7)[6k, n], (53)[6k, m ,n]; chalk and prefatigue effects (102)[1e]<br>**No data on quality criteria reported:** (33)[1d], (31)[4i]<br>**Diagnostics literature: reliability:** inter-rater: r=0.93-0.99 (nv); very high level of standardization; r=0.87-0.97 (nv) (181)						

Supplementary Table 16. Implementation variations, objects of measurement, measured values and units, data on quality criteria, and further information on pinching a dynamometer.

**pinch a dynamometer**<br>0/90, – | 90, 90/0 | –/0/– | –<br>sitting | standing | –<br>I/II | I/III | I/II-III | I/II-IV | I/II-V | –<br>unilateral (both sides/dominant only) | –	
**Object of measurement:**<br>measured value (unit)	**pinch / pincer isometric MS**<br>maximum force applied to the device (N) or (kg)
**Reliability**	*inter-session (8wk)*<br>**inter-adv. m/f:** CV=24-30% (107)<br>*intra-session*<br>**inter.-elite m:** r=0.99 ($CI_{95\%}$=0.98-0.99) - one measurement, r>0.99 ($CI_{95\%}$=0.99-0.99) – mean (108)
**Validity**	**inter-adv. m/f:** CCC=0.99 ($CI_{95\%}$:0.99-0.99) (107)<br>**inter-adv. m:** r=0.54-0.56*; c >nc* (109)<br>**inter-higher elite m/f:** r=0.442* (110)<br>**nc-lower grade:** ns diff. (97)<br>**lower grade m:** sig. higher scores in better c* (3)<br>**lower grade-adv.: m:** r=0.59***; **f:** r=0.22 (ns) (95)<br>**lower grade-adv. m/f:** r^2=0.59*** (63)<br>**inter.-elite: m:** r=0.27 (ns), **f:** r=0.41 (ns) (94)<br>**~inter.: m>f***** (24)<br>**~inter.:** corr. betw. top-roped climbing time and test ratios **m:** r= -0.937***, **f:** r= -0.774** (24)
**Additional data reported:** training effects (107)<br>**No data on quality criteria reported:** (102)	

Supplementary Table 17. Implementation variations, objects of measurement, measured values and units, data on quality criteria, and further information on gripping a dynamometer.

<table>
<tr><td colspan="6">grip a hand grip dynamometer
opposing thumb grip
-/90/- | 0/90/- | 0/90/45 | -/90/15 | 0/0/- | 0/0/slightly adducted
forearm medially rotated | hand supinated | forearm supported or not
sitting | standing
as strong and fast as possible | maintain 80 or 50% of MVC | 1 MVC (2, 3, 5s) | 3, 10 or 20 intermittent MVCs (5:5s or x:3s)
unilateral both hands | dominant hand only | non-dominant hand only | right hand only</td></tr>
<tr><td>Object of measurement: measured value (unit)</td><td>hand isometric MS
maximum force (kg and N)[1]
mean maximum force (kg)[2]</td><td>hand isometric ES
RFD (N/s)[3]</td><td>hand isometric MS + ES
maximum force (N)[4]
$RFD_{95\%, 500, 100, 200 ms}$ (Ns)[5]</td><td>hand isometric ME
oxidative capacity in dominant forearm flexor: half time recovery of tissue saturation index (s)[6]
time to failure (s)[7]
MMG (Hz)[8]
EMG (Hz)[9]
variation coefficient (#)[10]</td><td>hand intermittent isometric MSE
fatigue index (# and %)[11]
maximum force (N)[12]</td></tr>
<tr><td>Reliability</td><td>intra-session
lower grade-elite: ICC=0.97 (117)[1]
lower grade-elite no data: ICC<0.97 (4)[1]
inter. and elite m/f: ns diff. betw. ri and le hand (113)[1]

inter-session
2d elite m: ICC=0.975 ($CI_{95\%}$=0.84-0.99) (80)[1]
same day adv. f/m: ICC=0.912*** (112)[1]
7d adv.-elite m: CV=33.0 (83)[1]

intra-tester
lower grade m/f: ICC=0.88 (118)[1]</td><td>inter-session
7d adv.-elite m: CV=24.7-30.9 (83)[3]</td><td>inter-session
no data lower grade-elite m: RFD_{200ms} highly reliable, CV=11.78% while for other RFD CV>14% (115)[5]
4 wks elite-higher elite m: ICC= 0.83-0.98, CV=4-6% (119)[5]
intra-session
lower grade-elite m: ICC=0.94-0.99, CV=9.81-22.96% (115)[5]</td><td>inter-session
7d adv.-elite m: CV=33.0 (83)[7]</td><td>inter-session (7d)
lower grade-elite no data: ICC=0.93, CV=3.2% (4)[12]</td></tr>
<tr><td>Validity</td><td>lower grade-elite m/f: sig. correlation with performance (nd), ns diff. betw. lower grade and adv. (117)[1]
lower grade-higher elite m/f: correlation with climbing volume and experience (nd) (84)[1]
no data m: higher than nc*(111)[1]
elite m: higher than nc* (120)[1]
inter.-adv. m>nc*, ns correlation with climbing experience (109)[1]
nc-lower grade ns diff. in f (97)[1], and in m (3)[1]
no data m/f: m>f*** , ns corr. with climbing time (R=0.11) (121)[1]
adv.-elite m/f: ns correlation (122)[1]
elite-higher elite m: ns correlation (38)[1]
inter-adv. m: r=0.56*** ; f: r=0.60*** (95)[1]
lower grade-adv.: m: R=0.53 (nv), f: R=0.72 (nv) (123)[1]
nc<inter. m (nd) (124)[1]
nc-adv. m/f: ns diff. (125)[2]
inter.-elite: m: r=0.34-0.53 (ns), f: r=-0.1-0.14 (ns) (94)[1]</td><td>-</td><td>lower grade.-elite m: diff. betw. grip types (nv)**[4]; elite>adv and lower grade (nd)[5] (115)</td><td>inter.-adv. m: inversely associated with performance** (β=-0.659, $CI_{95\%}$=-0.946- -0.232) (45)[6]
elite m>nc*[7, 8, 9], elite<nc throughout first 20s*[10] (120)
no data m>nc*[7], no data<nc throughout first 20s*[10] (111)
lower grade- elite m/f: r^2=0.59*** (6)[7]</td><td>lower grade-elite no data: eff. of expertise*** (η^2=0.42, 1-β=0.99), diff. betw. all levels* (4)[11]
inter.-adv. m/f: r=-0.60** (62)[11]</td></tr>
</table>

	**inter.<elite m/f** (nd) (113)[1]<br>**adv.-elite m/f:** ns diff. betw. semi-finalists and finalists, **f:** finalists<semi-finalists (nv), semi-finalists **m>f** (nv) (126)[1]<br>**lower grade-elite no data:** ns tendency with expertise* (4)[1]<br>**adv. m>nc*** (96)[1]<br>**inter.-elite m:** R=0.56-0.57** (73)[1]<br>**adv.-elite m/f:** ns diff. (47)[1]<br>**lower grade<inter. m*** (3)[1]<br>**inter.-adv.* and elite-higher elite>nc m*****, **inter-higher elite m:** r=0.35* (99)[1]<br>**inter.: m>f*****, corr. with climbing time **m:** r =-0.96***, **f:** r= -.88*** (24)[1]<br>**inter.-elite m:** ns correlation (85)[1]<br>**lower grade-elite m/f:** r^2=0.59*** (6)[1]<br>**inter.-adv. m:** ns diff. (98)[1]<br>**no data m:** ns diff. (48)[1]				

Additional data reported: training effects (34, 80, 112)[1], (56, 57)[2], (119)[4,5]; recovery effects (65, 127)[1]; climbing effects (61, 73, 113)[1]; fatigue effects (128)[1], supplementation effects (46)[1]
No data on quality criteria reported: (75, 114,116, 122, 129)[1]
Diagnostics literature: maximum hand grip force: reliability**:** very high inter-rater reliability and level of standardization; r= 0.89-0.96 (nv); validity: construct and content validity can be considered as given, high correlation betw. different tests, no specialized age group or gender (97)

Supplementary Table 18. Implementation variations, objects of measurement, measured values and units, data on quality criteria, and further information on applying force on a hold.

**apply force on hold**<br>1 MVC as fast and hard as possible [1] | maintain 20-25, 40, 50, 60, 80 % of MVC [2] | intermittent 40, 60, 70, 80, 100 % of MVC (5:3, 5:5, 7:3, 6:4, 8:2, 10:10, 18:12 s) [3] | 100 % MVC for 2, 3-6, 30 s [4]<br>90/90/20 | 90/90/30 | 90/90/60 | 90/90/45 | 90/90/0 | 90/90/- | 180/0/- | 170-180/sb/- | -/50/130 | preferred angle<br>slope crimp | half crimp | open crimp | pinch | jug | sloper<br>2.8 | 4.3 | 5.8 | 7.3 | 8 | 10 | 12 | 20 | 22 | 23 mm<br>4 fingers | III-IV | II-III<br>unilateral both arms tested | left side only | right side only | dominant side only | bilateral<br>sitting | crouching | standing | hanging | leaning over<br>elbow supported | elbow not supported					
**Object of measurement:** measured value (unit)	**finger isometric ES+MS**<br>maximum force (N)[a]<br>$RFD_{95\%, 50, 100, 200\ ms}$ (N/s)[b]	**finger isometric (intermittent) ME**<br>time to failure (s)[c]<br>number of repetitions (#)[d]<br>force time integral (Ns)[e]<br>critical force (%)[f]<br>$blood\ pressure_{30\ s\ post}$ (mmHg)[g]<br>blood flow (ml/min)[h]<br>$lactate_{1, 3, 5, 7, min\ post}$ (mmol/L)[i]<br>oxidative capacity index (%)[j]<br>muscle blood volume (#)[k]<br>time to half recovery (s)[l]<br>muscle oxygenation (changes) and oxidative capacity in FDP and FCR (%)[m]<br>EMG (Hz)[n]<br>fatigue index (#)[o]<br>VO2 (L/kg)[p]<br>RER (#)[q]	**finger isometric (intermittent) MSE/CF**<br>maximum force (N)[r]<br>number of repetitions (#)[s]<br>EMG (Hz)[t]<br>fatigue index (# or %)[u]<br>critical force (N)[v]<br>impulse above critical force (kg·s)[w]	**finger isometric (intermittent) MS**<br>maximum force /weight held (kg or N)[x]<br>maximum force applied/weight held for 2-5 s (kg or N)[y]<br>number of repetitions (#)[z]<br>EMG (Hz)[aa]	**finger+wrist concentric-eccentric MS**<br>maximum force (N)[ab]
**Reliability**	*inter-session*<br>*7-14 d* **nc-elite no data:** CV=0.56-0.94*** (η^2=0.31-0.92), r>0.80 (nv): dominant hand[a,b 95%, 200ms], both hands[b 50, 100, 200 ms], r<0.60 (nv): non-dominant hand [b 200 ms], 0.60<r<0.80 (nv) other variables (140)[1]<br>*7d* **adv.-elite m:** ICC=0.21 [1b], ICC=0.94 [1a] (130)<br>*intra-session*<br>**elite no data:** ICC=0.74-0.90, CV=7.77-16.22%; **inter.-adv.** ICC=0.58-0.98, CV=9.83-28.34%, **nc** ICC=0.40-0.91, CV=12.56%-25.44% (140)[1b]<br>**elite no data:** ICC=0.95-0.98, CV=2.90-4.33% ; **inter-adv.** ICC=0.94-0.99,	*sustained*<br>*intra-session*<br>**lower level-adv. m:** CV=0.5% (141)[2e, l, m]<br>**elite m:** ICC=0.85-0.92 (138)[2c, e, i, p, q]<br><br>*intermittent*<br>*intra-session*<br>**elite m:** ICC=0.85-0.92 (138)[3c, e, i, p, q]<br>*inter-session*<br>*7d* **adv.-elite m:** ICC=0.89[3c], ICC=0.91[3e], ICC=0.85[3d] (130)<br>*3 sessions in 4 wks* **inter.-elite m/f:** ICC=0.29-0.69, CV=8.3-41.8% (110)[3m]<br>*7d* **lower grade-adv. m/f:** CV<2.5% (142)[3c, e, n]<br>*4 sessions in 4 wks with at least 1 day in betw.* **adv.-elite m:** 1 visit is a reliable measure (nd) (93)[3f]	*sustained*<br>*inter-session*<br>***7d*** **adv.-elite m:** ICC=0.92-0.94 (130)[4r]<br>*intra-session*<br>**elite m:** ICC=0.85-0.92 (138)[4r, u]<br><br>*intermittent*<br>*inter-session*<br>***no data*** **no data:** ICC=0.96 (9)[3v], ICC=0.87 (132)[3w]	*sustained*<br>*inter-session*<br>*6-7d* **lower grade-higher elite m/f:** r=0.88-0.97 (nv) (136)[4x]<br>*1d* **inter.-adv. m:** r=0.98-0.99*** (143)[4x]<br>*7d* **adv.-elite m:** ICC=0.88 (130)[4x]<br>*2d* **inter-higher elite m/f:** ICC>0.91 (144)[4x]<br>*3 sessions in 4 wks* **lower grade-adv. m/f:** CV<2.5% (142)[4x]<br>*same day* **nc-adv. m:** ICC=0.92**, CV=2.2 % (163)[4x]<br>**inter-adv. m:** CV=0.5% by Fryer et al. 2014 (145), (141)[4x]<br>*intra-session*<br>**lower grade-higher elite m/f:** r=0.88-0.94 (nv) (136)[4x]<br>**lower grade-elite m/f:** ICC=0.97-0.98 (92)[4y]<br>**inter.-adv. m/f:** R=0.92-0.95 (nv) ($CI_{95\%}$=0.891-0.975), ns diff. betw. trails (146)[4x]<br>**no data:** Cronbach's alpha=0.99 (nv) (110)[4x]	-

	CV=2.64-5.93%; **nc** ICC=0.89-0.92, CV=5.46-9.99% (140)[1a]<br>**adv.-elite m:** ICC>0.90 (9)[1a, b]; ICC=0.94[1b] (130)				
**Validity**	**adv.-elite b m>elite c m*** (9)[1a, b], r=0.65-0.82* (130)[1a]	*sustained*<br>**inter.-elite m/f:** r=0.35 (ns) (110)[2e]<br>**adv.-elite>nc*, m>f*** (147)[2e]<br>**lower level-adv. m:** ns diff. [2e], betw.-group diff. for FDP and FCR*** [2m], ** [2l] (141)<br>**lower grade-inter. m:** ns diff. to rowers or leg trained athletes (148)[2c, g, i]<br>**inter.-adv.>nc*** (109)[2c]<br>**nc-no data m:** ns diff. (48)[2c]<br>**inter.-adv. m:** R^2=0.56 (nv)[2c, e, m], d=0.94*[c], d=1.47***[2e], d=0.44 (ns)[2m] (41)<br>**inter-.elite no data:** ns diff. betw. b and c [2m], b<c (ns)[2c] (149)<br>**elite m:** r=0.72** (138)[2e]<br>**elite-higher elite m:** r=-0.26 (ns)[2c], r=0.02 (ns)[2e], **higher elite>elite***[2e] (156)<br><br>*intermittent*<br>**inter.-adv. m:** R^2=0.43 (nv)[3c, e, m], d=0.51 (ns)[3c], d=0.07-0.33 (ns)[3m] (41)<br>**elite-higher elite m:** ns diff.[3c, e, o], r=-0.09-0.19 (ns)[3e], r=-0.27 (ns)[3e], r=-0.06 (ns)[3o] (156)<br>**adv. m:** c>b and nc*[3c],c and b>nc*[3j] (150)<br>**inter.-adv. m:** adv.>inter.* and nc* [3e, m] (deoxygenation), ns diff.[3e, m (reoxygenation)], adv.>nc* but not inter.[3h] (145)<br>**adv.-elite>nc***[3c], *[3m (reoxygenation)] (147)<br>**inter.-adv. m: adv.>nc***[3e, m (reoxygenation)], ns diff.[3c, m] (141)<br>**inter.-adv. m:** ns diff[3c, g], c>nc (nv) [3e,] ns relationship with performance[3c] (2)<br>**inter-adv. m/f:** ns diff. betw. b and c (101)[3c]<br>**lower grade-inter. m:** ns diff. to rowers or leg trained athletes and among climbers (148)[3c]	*sustained*<br>**elite m:** r=0.82*** (138)[4r]<br>**higher elite>elite m****, r=0.80** (156)[4r]<br>**adv.-elite m:** corr. with climbing- (r^2=0.42, nv) and bouldering ability (r^2=0.58, nv) (130)[4r]<br><br>*intermittent*<br>**adv. m>nc*** (96)[4r]<br>**inter.-higher elite m/f:** R^2=61% (nv) (sport climbing)[3v], R^2=26% (nv) (bouldering)[3v]; R^2=34% (nv) (bouldering)[3w] (132)<br>**inter.-adv.>nc**, elite-higher>nc***, inter-adv.<elite-higher elite***** r=0.60*** (199)[3r]<br>**adv.-elite m/f:** ns diff. (47)[3u]	*sustained*<br>**lower grade-elite m/f:** ns corr. with performance (14)[4y]<br>**no data m/f:** ns diff. betw. age groups (54)[4y]<br>**no data m/f:** proved to be empirically and statistically relevant to performance in at least one of the competition disciplines of sport climbing (32)[4y]<br>**adv.-elite m/f:** r=small-moderate (nv) (15)[4r]<br>**lower grade-higher elite m/f:** r=0.79-0.81 (nv) (136)[4x]<br>**elite<higher elite m****, r=0.80** (156) [4x]<br>**adv.-elite m:** corr. with climbing- (r^2=0.48, nv) and bouldering ability (r^2=0.66, nv) (130)[4x]<br>**elite m:** r=0.81*** (138)[4x]<br>**elite-higher elite m:** ns correlation (38)[4x]<br>**adv.-elite m:** r=0.63* (173)[4x]<br>**inter.-adv. m:** R^2=0.30 (nv) (41)[4x]<br>**inter. and adv.<elite f***, R^2=0.16*-0.53** (134)[4x]<br>**inter-higher elite m/f:** r=0.42–0.50 (nv) (144)[4y]<br>**lower grade-inter. m>**rowers** and aerobically leg trained athletes*** (148)[4x]<br>**nc<inter. f***, **inter.>lower grade***, **nc-lower grade:** ns diff. (97)[4x]<br>**inter-.elite no data:** b>c* (149)[4x]<br>**inter-higher elite m:** r=0.60*** (99)[4x]<br>**adv.-elite>nc**, m>f*****, r^2=0.84** (147)[4x]<br>**inter-adv. m:** r=0.26*; **f:** r=0.19 (ns) (95)[4x]<br>**inter-higher elite m/f:** r=0.39* (110)[4x]<br>**inter.-adv. m:** r=0.71 (nv) (2)[4x]<br>**adv. m>nc***** (196)[4x]<br>**lower grade-inter.:** corr. to climbing time **m:** r=-0.96- -0.80**, **f:** r=-0.88- -0.77**; **m>f**** (24)[4x]<br>**nc and adv.-elite no data:** b>c* and nc, c>nc* (8)[4x]<br>**adv. m:** c and b>nc*, b>c* (150), (141)[4x]<br>**inter-adv. m>nc*, adv.>inter*** (141)[4x]<br>**inter.-adv f:** r=0.44-0.64 (131)<br><br>*intermittent*<br>**adv.-elite no data:** ns diff. [3x], **elite> adv.****[3z] (29)	**adv.-elite m:** concentric wrist flexion: r=0.57*** (133)[4ab]
**Additional data reported:** training effects (90)[2c, j], (7)[3c, 4x,]; blood flow restriction effects (105)[3c] ; recovery effects (151)[3c, e, h, j, 4x]; fatigue effects (152)[4x], (2)[2e, g, c, j, k], (153)[4x]; arm position effects (130)[1a,b, 4r], (187)[4y]; side of measurement effects (109)[2c]; hold type effects (136)[4x], (137)[4x], (143)[4x], (139)[4x], (29)[3a]; injury effects (154)[4x]<br>**No data on quality criteria reported:** (106, 1135 151)[4x], (30)[3c], (132)[3v], (155)[2c, e, m]<br>**Diagnostics literature/other studies:** excellent test-retest reliability (nd), small within-subject test-retest variation, CV=6.8%, small change in test-retest group mean (typical error 15.3N, 5.5%), test-retest corr. (r=0.91, ICC=0.94** (173)[3v]					

Supplementary Table 19. Implementation variations, objects of measurement, measured values and units, data on quality criteria, and further information on the power-slap test.

**power-slap test**<br>reach and slap with one hand [1] | reach and slap with both hands [2] | reach and hold with one hand [3]<br>one time | 10 times with 10 s rest		
**Object of measurement:**<br>measured value (unit)	**upper limb concentric ES**<br>maximum height slapped (cm)[a]<br>maximum height slapped relative to arm span (%)[b]<br>highest rung reached and held for 2s (#)[c]	**upper limb concentric ESE**<br>fatigue index (#)[d]
**Reliability**	*inter-session:*<br>*2-7d and >7d* ICC=0.95-0.98 (14, 158)[1a]<br>- ICC=0.98 (112)[1a]<br>*7d* ICC=0.98 (157)[2a]<br><br>*intra session:*<br>ICC=0.98 (157)[2a]	-
**Validity**	**lower grade-elite no data:** correlation to rel. upper limb power r=0.70**, b>c*** (157)[2a]<br>**no data m/f:** older>younger** (54)[1a]<br>**no data m/f:** proved to be empirically and statistically relevant to performance in at least one of the competition disciplines of sport climbing (32)[1a, b]<br>**elite>inter.-adv. f:** sig. associated performance* (134)[1a]<br>**lower grade-elite m/f:** r=0.69-0.73 (nd) (14)[1a]<br>**lower grade-elite m/f:** r=0.69-0.73***, **elite> inter.**** and **lower grade*****, **adv.>inter.** (le***) and **lower grade***** (158)[1a]	**lower grade-elite no data:** eff. of expertise***<br>(η^2=0.69, 1-β=0.99), diff. betw. lower grade and other levels (nd)* (4)[2d]
**Additional data reported:** training effects (53)[3c], (112)[1a], effect of grip width (158)[1a]<br>**No data on quality criteria reported:** (31)[-]		

Supplementary Table 20. Implementation variations, objects of measurement, measured values and units, data on quality criteria, and further information on the medicine ball throw.

**medicine ball throw**<br>lying[1]/sitting[2]	
**Object of measurement:**<br>measured value (unit)	**upper limb ES**<br>maximum distance (cm)
**Reliability**	*inter-session (same day)*<br>**adv. f/m:** ICC=0.96*** (112)[2]
**Additional data reported:** training effects (112)<br>**No data on quality criteria reported:** (31)<br>**Diagnostics literature:** standing, high level of standardization, inter-rater reliability r=0.90 (nv), correlation with judgement of an expert r=0.46 (nv) and sig. correlation with self-evaluation and sport grade(in children aged 6-11, nd) (181)	

Supplementary Table 21. Implementation variations, objects of measurement, measured values and units, data on quality criteria, and further information on the bicep strength test.

**biceps strength (no further explanation)**	
**Object of measurement:**<br>measured value (unit)	**biceps MS**<br>maximum force (N)
**Validity**	**inter-adv. m:** r=0.45***, **f:** r=0.29 (ns) (95)

Supplementary Table 22. Implementation variations, objects of measurement, measured values and units, data on quality criteria, and further information on the shoulder strength test.

**shoulder strength test**<br>internal and external rotation | extensions		
**Object of measurement:**<br>measured value (unit)	**shoulder concentric-eccentric MS**	**shoulder concentric MS**<br>maximum force (kg)[1]<br>work out-put (J)[2]
**Validity**	**nc-no data m/f:** c conventional work ratios<nc; c functional work ratio of ecc ER:con IR<nc; c functional work ration of ecc IR:con ER>nc. (160)	**lower grade-elite m/f:** r^2=0.59*** (6)

Supplementary Table 23. Implementation variations, objects of measurement, measured values and units, data on quality criteria, and further information on the elbow strength tests.

**elbow strength tests**<br>internal and external rotation | extensions	
**Object of measurement:**<br>measured value (unit)	**elbow concentric MS**<br>maximum force (kg)
**Validity**	**inter.-elite m/f:** r=0.44-0.63 (159)

Supplementary Table 24. Implementation variations, objects of measurement, measured values and units, data on quality criteria, and further information on push-ups.

**push ups**<br>as many as possible in 15 s	
**Object of measurement:**<br>measured value (unit)	**upper limb ESE**<br>-
**No data on quality criteria reported:** (29)<br>**Diagnostics literature:** push up, lie down, clap behind back, upper limb and core ME, number of repetitions (#): reliability: high level of standardization, inter-rater: r=0.86-0.98(nv) (r=0.84-0.95, nv (172)); inter-session r=0.69-0.79 (nv) (r=0.71-0.99, nv (172)), validity: expert ratings for test items=1.31-2.5, criteria validity proven (97)	

Supplementary Table 25. Implementation variations, objects of measurement, measured values and units, data on quality criteria, and further information on campus board performance.

**campus board performance**<br>laddering | single reaches with feet footholds	
**Object of measurement:**<br>measured value (unit)	**upper limb ESE**<br>number of repetitions (#)
**Additional data reported:** training effects (39, 53)	

Supplementary Table 26. Implementation variations, objects of measurement, measured values and units, data on quality criteria, and further information on the arm jump test.

**arm jump test**<br>with countermovement [1] | without countermovement [2]<br>reach and hold with both hands	
**Object of measurement:**<br>measured value (unit)	**upper limb (eccentric)-concentric ES**<br>impulse from onset to contact loss (ns)[a]<br>maximum force (N)[b]
**Reliability**	*inter-session (2d)*<br>high to very high (nd) (161)[1a, 1b, 2a, 2b]

Supplementary Table 27. Implementation variations, objects of measurement, measured values and units, data on quality criteria, and further information on the bench press.

**bench press**<br>1RM	
**Object of measurement:** measured value (unit)	**upper limb concentric ES+MS**<br>velocity (m/s)[1]<br>maximum force (N)[2]
**Validity**	**lower grade-elite no data:**<br>performance no effect on velocity (4)[1]

Supplementary Table 28. Implementation variations, objects of measurement, measured values and units, data on quality criteria, and further information on the pull down.

**pull down**	
**Object of measurement:**<br>measured value (unit)	**upper limb concentric-eccentric MSE**<br>12 RM (kg)
**No data on quality criteria reported:** (64, 65)	

Supplementary Table 29. Implementation variations, objects of measurement, measured values and units, data on quality criteria, and further information on the traction test.

**traction test**		
**Object of measurement:**<br>measured value (unit)	**upper limb concentric ES**<br>mean power (W)[1]	**upper limb concentric-eccentric ME**<br>mean power (W)[2]<br>number of cycles completed (#)[3]
**Validity**	**higher elite m:** possible statistical correlation with performance (105)[1]	-
**No data on quality criteria reported:** (139)[2,3]		

Supplementary Table 30. Implementation variations, objects of measurement, measured values and units, data on quality criteria, and further information on rowing ergometry.

**rowing ergometry**<br>rowing machine [1] | climbing hold attached to rowing ergometer [2]		
**Object of measurement:**<br>measured value (unit)	**upper limb concentric-eccentric E**<br>peak power (W)[a]<br>time to failure (s)[b]<br>maximum VO2 (ml/min/kg)[c]<br>HR (bpm)[d]<br>lactate (mmol/L)[e]<br>RER (#)[f]<br>RCP (%)[g]	**upper limb concentric MS**<br>1 RM (kg)[h]<br>EMG (Hz)[i]
**Reliability**	-	*inter-session (2d)*<br>**inter.-adv.:** ICC=0.79-0.85 ($p \leq 0.01$) (163)[2h]
**Validity**	**adv.-elite m:** r=.85[1c], corr. (nd)**[1a] (162)	**inter.-adv. m>nc****[2h], r=0.72-0.73*[2h], **[2i (flexor digitorum superficialis)], inter.-adv. ns diff. from nc[2i (posterior deltoid)] (163)

Supplementary Table 31. Implementation variations, objects of measurement, measured values and units, data on quality criteria, and further information on arm crank ergometry.

**arm crank ergometry**<br>consistent | incremental	
**Object of measurement:** measured value (unit)	**upper limb concentric-eccentric endurance**<br>maximum and average power (W)[1]<br>maximum force (N)[2]<br>maximum VO_2 (ml/kg/min)[3]<br>time to failure (s)[4]<br>$RCP_{1,2}$ (ml/min/kg)[5]<br>HR (bpm)[6]<br>expiratory ventilation (%)[7]
**Validity**	**inter-adv. m:** r=0.56***, **f:** r=0.20-0.28 (ns) (95)[1]<br>**nc<inter-adv. no data***[3], **inter.-adv.** ns diff.[3], **adv.>inter.***[4] and **nc*****[4], **inter.-**nc ns diff.[4], **adv.>nc** for RCP_1*;**adv.-inter.** or **inter. vs. nc**, ns diff. regarding performance for RCP_2[5] (165)<br>**nc-no data m:** ns diff. (48)[3, 6, 7]
**Additional data reported:** individual differences (30)[2]<br>**Diagnostics literature**: intra-rater reliability: ICC=0.82; inter-session: ICC=0.76; validity: correlation with VO2max on bicycle ergometer ICC=0.64 (182)[1]	

Supplementary Table 32. Implementation variations, objects of measurement, measured values and units, data on quality criteria, and further information on the shoulder flexibility test.

**shoulder flexibility test**<br>raise rod overhead and behind back with straight arms [1]	
**Object of measurement:**<br>measured value (unit)	**shoulders active dynamic FLEX**[1]<br>minimum distance between hands (cm)
**Validity**	**inter.-elite f:** ns diff. (134)
**Diagnostics literature:** scaled rod over the head, measure of shoulder FLEX: reliability: inter-rater: r=0.91-0.98 (nv); intra-session: r=0.88-0.98 (nv); validity: valid for all age groups (nd) (183)	

Supplementary Table 33. Implementation variations, objects of measurement, measured values and units, data on quality criteria, and further information on the shoulder abduction and flexion test.

**shoulder abduction and flexion**<br>maximum range of abduction and flexion	
**Object of measurement:**<br>measured value (unit)	**shoulders active static FLEX**[2]<br>maximum range of motion (°)
**Validity**	**lower grade-adv. m/f:** r^2=0.02 (ns) (6)

Supplementary Table 34. Implementation variations, objects of measurement, measured values and units, data on quality criteria, and further information on the lower limb strength test.

**lower limb strength test**<br>flexion and extension	
**Object of measurement:**<br>measured value (unit)	**lower limb concentric MS**<br>maximum force (lbs)
**Validity**	**lower grade-elite m/f:**<br>r^2=0.59*** (6)

Supplementary Table 35. Implementation variations, objects of measurement, measured values and units, data on quality criteria, and further information on the jump with high foot.

**jump with high foot**	
**Object of measurement:**<br>measured value (unit)	**lower limb concentric ES**<br>jump height (cm)
**Reliability**	*intra-session*<br>**adv.-elite m/f:** r=0.76-0.92 (nv) (15)<br><br>*inter-session (7d)*<br>**adv.-elite m/f:** inacceptable reliability (15)
**No data on quality criteria reported:** (32)	

Supplementary Table 36. Implementation variations, objects of measurement, measured values and units, data on quality criteria, and further information on the counter movement jump.

**counter movement jump**	
**Object of measurement:**<br>measured value (unit)	**lower limb eccentric-concentric ES**<br>vertical jump height (cm)[a]<br>power during the jump (W)[b]
**Validity**	**no data m/f:** proved to be empirically and statistically relevant to performance in at least one of the competition disciplines of sport climbing (32)[a]<br>**inter.-elite f:** ns diff. (134)[a]<br>**adv.-elite m/f:** ns diff. (47)[a]<br>**elite-higher elite m:** cor. with climbing time r=-0.79[a] - -0.75[b] (nv) (79)

Supplementary Table 37. Implementation variations, objects of measurement, measured values and units, data on quality criteria, and further information on the squat jump.

**squat jump**<br>hands on hips [1] | arm swing allowed [2]	
**Object of measurement:**<br>measured value (unit)	**lower limb concentric ES**<br>vertical jump height (cm)
**Validity**	**adv.-elite m/f:** ns diff. (47)[1]<br>**no data m/f:** proved to be empirically and statistically relevant to performance in at least one of the competition disciplines of sport climbing (32)[1]<br>**inter.-elite: m**: r=0.23 (ns), **f:** r=0.33 (ns) (94)[2]

Supplementary Table 38. Implementation variations, objects of measurement, measured values and units, data on quality criteria, and further information on the standing long jump.

**standing long jump**	
**Object of measurement:**<br>measured value (unit)	**lower limb eccentric-concentric ES**<br>distance jumped (cm) or (m)
**Additional data reported:** training effects (33)<br>**No data on quality criteria reported:** (31)<br>**Diagnostics literature:** reliability: very high level of standardization, inter-rater: r=0.86-0.99 (nv) (0.88-0.94, nv (172)), inter-session: r=0.52-0.92 (nv) (0.65-0.96, nv (172)), criteria validity proven, expert ratings on test items=1.31-2.5 (181)	

Supplementary Table 39. Implementation variations, objects of measurement, measured values and units, data on quality criteria, and further information on the vertical jump.

**vertical jump**	
**Object of measurement:**<br>measured value (unit)	**lower limb ES (contraction unclear)**<br>vertical jump height (cm)
**Validity**	**no data m/f:** older>younger** (50)
**Additional data reported: -**<br>**No data on quality criteria reported: -**<br>**Diagnostics literature:** jump and reach test: reliability: very high level of standardization, inter-rater: r>0.72 (nv) (r=0.87-0.97, nv (172); inter-session: r=0.87 (nv) (r=0.60-0.98, nv (172); validity: valid for subjects older than 6 (m and f) (nd) (181)	

Supplementary Table 40. Implementation variations, objects of measurement, measured values and units, data on quality criteria, and further information on one legged squats.

**one legged squat**	
**Object of measurement:**<br>measured value (unit)	**lower limb concentric-eccentric ME**<br>number of repetitions (#)
**No data on quality criteria reported:** (113)<br>**Diagnostics literature:** reliability: inter-rater r=0.90-0.96 (nv) for ages 10-18 (185)	

Supplementary Table 41. Implementation variations, objects of measurement, measured values and units, data on quality criteria, and further information on treadmill running.

**treadmill running**<br>incremental increase in speed [1] | incremental increase in elevation [2]	
**Object of measurement:**<br>measured value (unit)	**lower limb endurance**<br>time to failure (s)[a]<br>slope (%)[b]<br>velocity (km/h)[c]<br>peak HR (bpm)[d]<br>maximum VO_2 (L/min) or (ml/min/kg)[e]<br>VT (L)[f]<br>VE (L/min)[g]<br>RER (#)[h]<br>RCP (%)[i]<br>fR (brpm)[j]<br>lactate (mmol/L)[k]
**Validity**	**inter.-adv. m:** ns association with performance (41)[2e]<br>**adv.-elite m:** ns corr. with performance [1e], corr. to weekly training hours (r=80**)[1e], ns corr. with performance[1a, c, d, h, i, k] (162)<br>**inter-adv. m:** r=0.28**; **f:** r=0.17 (ns) (95)[2e]<br>**inter.-adv. m:** d=0.43 (ns)[2a], d=0.24 (ns)[2b], d=1.23**[2d], d=0.23 (ns)[2d], d=0.17 (ns)[2f], d=0.39 (ns)[2h], d=0.55 (ns)[2j], **inter>adv.**[**2d] (41)
**Additional data reported:** no reliable intensity indicators (nd) (162, 164)[1d, k]<br>**No data on quality criteria reported:** (37)[1a, d, e, g]<br>**Diagnostics literature:** reliability: r= 0.75-0.99 (186)[e, k]	

Supplementary Table 42. Implementation variations, objects of measurement, measured values and units, data on quality criteria, and further information on cycle ergometry.

**cycle ergometry**<br>discontinuous incremental [1] | consistent (Wingate test) [2]	
**Object of measurement:**<br>measured value (unit)	**lower limb E**<br>HR (bpm)[a]<br>fR (brpm)[b]<br>V_T (L)[c]<br>VO_2 and VCO_2 (ml/min/kg)[d]<br>RER (#)[e]<br>velocity (m/min)[f]<br>lactate (mmol/L)[g]<br>mean and peak power (W)[h]<br>power decline (%)[i]
**Validity**	**lower grade-elite m/f:** r^2=0.59*** (6)[2h, i]
**No data on quality criteria reported:** (42)[1a-g], (48) [c, d], (167)[a, d, e], (168)[d]	

Supplementary Table 43. Implementation variations, objects of measurement, measured values and units, data on quality criteria, and further information on the sit and reach test.

**sit and reach**<br>both legs straight [1] | one leg straight, one bent with foot on floor (back saver sit and reach) [2]	
**Object of measurement:**<br>measured value (unit)	**low back + hamstrings active static FLEX**<br>furthest distance reached (cm)
**Reliability**	*inter-session (7-14d)*<br>**lower grade-elite m/f:** ICC=0.97 ($CI_{95\%}$=0.92-0.99), MD=-0.03 (LoA=-2.59-2.53) (169)[1]
**Validity**	**m/f: elite>lower grade-adv.** (nv); ns corr. (nv) (169)[1]<br>**adv.-elite m/f:** ns diff. (47)[1]<br>**nc-inter. m:** ns diff. (3)[1]<br>**nc-inter. f:** ns diff. (97)[1]<br>**inter.-adv. m:** m: r=0.42**, f: r=0.17 (ns) (95)[1]
**Additional data reported:** training effects (114)[2]<br>**Diagnostics literature**: reliability: r>0.86-0.97 (nv), r>0.86 (nv); validity(aged 18-35): hamstrings (m: r=0.75, nv; f: r=0.66), nv, lower back (m: r=0.40, nv; :f r=0.25, nv) (183)[1]	

Supplementary Table 44. Implementation variations, objects of measurement, measured values and units, data on quality criteria, and further information on the lateral foot reach.

**lateral foot reach**	
**Object of measurement:**<br>measured value (unit)	**hip active static FLEX**<br>distance betw. foot and start foothold (cm)
**Reliability**	*inter-session (7-14d):*<br>**lower grade-elite m/f:** ICC=0.93 ($CI_{95\%}$=0.83-0.97), MD=-0.46 (LoA=-0.55-3.63) (169)
**Validity**	**lower grade-elite m/f:** r=0.24 (ns)-0.30*; ns diff. betw. ability groups (169)

Supplementary Table 45. Implementation variations, objects of measurement, measured values and units, data on quality criteria, and further information on the Grant foot raise.

**Grant foot raise**<br>with lateral hip movement [2] | without lateral hip movement [1]<br>toes 23 cm in front of a wall [a] | arms fully extended at 90° [b]	
**Object of measurement:**<br>measured value (unit)	**hip active static FLEX**<br>distance betw. ground and foot (cm)
**Reliability**	*inter-session (7-14d):*<br>**lower grade-elite m/f:** ICC=0.90 (CI95%=0.90-0.96), MD=-0.80 (LoA=-6.60-5.01)[1], ICC=0.93 ($CI_{95\%}$=0.84-0.97), MD=-0.82(LoA=-6.87-5.28)[2] (169)
**Validity**	**lower grade-inter. m:** ns diff. (13)[1a]<br>**nc-inter. f:** ns diff. (14, 97)[1a]<br>**inter< lower grade m/f***[1a,2a]; **higher ability c>lower grade-adv. (nv)** [1a,2a]; **lower grade-elite m/f:** r=0.20-0.23 (ns)[1a], r=0.31-0.34*[2a] (169)<br>**adv.-higher elite m:** ns corr. (nd) (170)[2a]<br>**inter-higher elite m/f:** r=0.250 (ns) (110)[2a]<br>**inter-adv. m:** r=0.07, (ns) **f:** r=0.24, (ns) (95)[-b]

Supplementary Table 46. Implementation variations, objects of measurement, measured values and units, data on quality criteria, and further information on the climbing specific foot raise.

**climbing specific foot raise**<br>with rotation (shoulders max break parallelism to wall) [1] | without rotation (shoulders remain parallel to wall) [2]	
**Object of measurement:**<br>measured value (unit)	**hip active static FLEX**<br>distance betw. foothold and foot (cm)[a]<br>knee position - knee height (cm, %)[b]
**Reliability**	*inter-session:*<br>*7-14d* **lower grade-elite m/f:** ICC=0.89 ($CI_{95\%}$=0.76-0.96), MD=-1.39 (LoA=-13.88-11.11) (169)[1a]<br>*7d* **adv.-elite m/f:** r=0.95-0.99 (nv) (15)[2b]
**Validity**	**elite>lower grade m/f*** and **inter.****; **lower grade-elite:** r=0.53-0.55* (1169)[1a]<br>**lower grade-elite m/f:** r=0.55*[1a]; ns diff. (14)[1a, 2a]<br>**no data:** empirically and statistically relevant to performance in at least one of the competition disciplines of sport climbing in m and f (32)[2a, 2b]<br>**adv.-elite:** corr. with **b** and **c** (r=0.88-0.95, nv), ns corr. with **sc** (15)[2b]

Supplementary Table 47. Implementation variations, objects of measurement, measured values and units, data on quality criteria, and further information on the hip abduction test.

**hip abduction with external rotation and hip flexion**	
**Object of measurement:**<br>measured value (unit)	**hip active static FLEX**<br>maximum range of motion (°)
**Validity**	**lower grade-elite m/f:** r^2=0.02*** (6)
**No data on quality criteria reported:** (131)	

Supplementary Table 48. Implementation variations, objects of measurement, measured values and units, data on quality criteria, and further information on the Draga test.

**Draga test**	
**Object of measurement:**<br>measured value (unit)	**hip active static FLEX**<br>distance betw. foot and calcaneal tuberosity (cm)
**Validity**	**adv.-higher elite m:** ns corr. (nd) (170)

Supplementary Table 49. Implementation variations, objects of measurement, measured values and units, data on quality criteria, and further information on the hip slide test.

**hip slide test**	
**Object of measurement:**<br>measured value (unit)	**hip active static FLEX**<br>distance betw. wall and hip (mm)
**Validity**	**inter-elite f:** ns diff. (134)

Supplementary Table 50. Implementation variations, objects of measurement, measured values and units, data on quality criteria, and further information on the foot loading flexibility test.

**foot loading flexibility test**	
**Object of measurement:**<br>measured value (unit)	**hip active static FLEX/climbing ability**<br>distance betw. start and end foothold (cm)
**Reliability**	*inter-session (7-14d):*<br>**lower grade-elite m/f:** ICC=0.96 ($CI_{95\%}$=0.89-0.98), MD=-0.12 (LoA=-4.57-4.82) (169)
**Validity**	**elite>lower grade-adv. m/f:** sig. diff. betw. groups ($F_{(3,42)}$=8.38)***: **elite>lower grade*****, **inter.***** and **adv.** (ns), **adv.> lower grade** and **inter.****; ns diff. betw. **lower grade** and **inter.; lower grade-elite m/f:** r=0.56-0.65* (169)

Supplementary Table 51. Implementation variations, objects of measurement, measured values and units, data on quality criteria, and further information on the asymmetry in reach test.

**asymmetry in reach test**	
**Object of measurement:**<br>measured value (unit)	**hip active static FLEX/climbing ability**<br>maximum reach (cm)[1]<br>symmetry index (#)[2]
**Reliability**	*intra-session:*<br>**no data m/f:** ICC=0.990-0.997 ($CI_{95\%}$=0.99-0.99); CV=1.31-2.53%[1];<br>ICC=0.89 ($CI_{95\%}$=0.77-0.95), CV=35.20%[2] (113)<br>*inter-session (no data):*<br>**no data m/f:** ICC=0.95-0.96 ($CI_{95\%}$=0.88-0.98), (CV=4.96-5.37%)[1];<br>ICC=0.87 ($CI_{95\%}$=0.72-0.94, CV=41.98%)[2] (113)

Supplementary Table 52. Implementation variations, objects of measurement, measured values and units, data on quality criteria, and further information on froggies.

**froggies**	
**Object of measurement:** measured value (unit)	**hip passive static FLEX** distance betw. wall and pubic symphysis (cm)
**No data on quality criteria reported:** (5, 30)	

Supplementary Table 53. Implementation variations, objects of measurement, measured values and units, data on quality criteria, and further information on the straddle test.

**straddle test** standing[1], sitting[2], or lying[3]	
**Object of measurement:** measured value (unit)	**hip + lower limb passive static FLEX** distance betw. ground and pubic symphysis[a]/ri medial calcaneus[b] distance betw. feet[c] angle betw. legs[d]
**Validity**	**adv.-elite m:** r=-0.48*[1a], r= -0.41*[2c] (170) **inter-elite f:** ns diff. (134)[1b] **nc-inter. m:** adv. sig. better than other groups* (3)[3c] **nc -inter. f:** ns diff. (97)[3c] **inter.-adv. m:** r=0.57***, **f:** r=0.16 (ns) (95)[-]
**Diagnostics literature:** reliability: inter-rater: r=0.73-0.81 (nv); inter-session (aged 6-18): r=0.73-0.97 (nv); validity: higher values standing compared to lying (172)[3c, 3d]	

Supplementary Table 54. Implementation variations, objects of measurement, measured values and units, data on quality criteria, and further information on the hip flexion and rotation test.

**hip flexion and rotation**	
**Object of measurement:** measured value (unit)	**hip active FLEX** maximum angle (°)
**No data on quality criteria reported:** (131)	

Supplementary Table 55. Implementation variations, objects of measurement, measured values and units, data on quality criteria, and further information on the leg flexion test.

**leg flexion test**	
**Object of measurement:** measured value (unit)	**lower limb active FLEX** maximum angle (°)
**No data on quality criteria reported:** (131)	

Supplementary Table 56. Implementation variations, objects of measurement, measured values and units, data on quality criteria, and further information on the super man.

**super man**	
**Object of measurement:** measured value (unit)	**core concentric-eccentric MS** distance between feet and fingertips (cm)
**Reliability**	*inter-session (3-10 d)* **adv.-elite m/f:** ICC=0.87 (86)
**Additional data reported:** training effects (86)	

Supplementary Table 57. Implementation variations, objects of measurement, measured values and units, data on quality criteria, and further information on momentum absorption.

**momentum absorption**	
**Object of measurement:** measured value (unit)	**core concentric MS** angle at first back sing (°)
**Validity**	**no data m/f:** r=-.01 – .31 (15) **adv.-elite m/f:** corr. with climbing- (r=0.74, nv) and boulder performance (r=0.65, nv) (15)

Supplementary Table 58. Implementation variations, objects of measurement, measured values and units, data on quality criteria, and further information on the core rotation test.

**core rotation test**	
**Object of measurement:**<br>measured value (unit)	**core concentric MS**<br>mean force held for 3s (N)
**Additional data reported:** training effects (86)	

Supplementary Table 59. Implementation variations, objects of measurement, measured values and units, data on quality criteria, and further information on the body lock off.

**body lock off**	
**Object of measurement:**<br>measured value (unit)	**core isometric ME**<br>time to failure (s)
**Reliability**	*inter-session (3-10 d)*<br>**adv.-elite m/f:** ICC=0.79 (86)
**Additional data reported:** training effects (86)	

Supplementary Table 60. Implementation variations, objects of measurement, measured values and units, data on quality criteria, and further information on the plank.

**plank**	
**Object of measurement:**<br>measured value (unit)	**core isometric ME**<br>time to failure (s)
**Validity**	**lower grade-elite m/f:** ns diff. (14)

Supplementary Table 61. Implementation variations, objects of measurement, measured values and units, data on quality criteria, and further information on the Sorensen test.

**Sorensen test**	
**Object of measurement:**<br>measured value (unit)	**core isometric ME**<br>time to failure (s)
**Validity**	**adv. m and nc:** ns diff. (96)<br>**lower grade-elite no data:** ns diff. (4)
**Diagnostics literature:** reliability: intra-tester ICC=0.79-0.80, inter-rater ICC=0.78; inter-session ICC=0.59 for patients with chronic back pain; validity: sig. corr. with decrease in EMG of various core muscles (r=0.47-0.71, nv) (187)	

Supplementary Table 62. Implementation variations, objects of measurement, measured values and units, data on quality criteria, and further information on the Kraus Weber test battery.

**Kraus Weber test battery**	
**Object of measurement:**<br>measured value (unit)	**core isometric ME**<br>test score (#)
**Validity**	**adv. m and nc:** ns diff. (96)

Supplementary Table 63. Implementation variations, objects of measurement, measured values and units, data on quality criteria, and further information on sit-ups.

**sit-ups**	
**Object of measurement:**<br>measured value (unit)	**core concentric-eccentric ME**<br>-
**No data on quality criteria reported:** (31)<br>**Diagnostics literature:** reliability**:** very high level of standardization: inter-session r=0.78-0.88 (nv), expert rating on test item core ME=1.75-2.17; validity: high construct and criteria validity (181)	

Supplementary Table 64. Implementation variations, objects of measurement, measured values and units, data on quality criteria, and further information on curl-ups.

**curl-ups**	
**Object of measurement:**<br>measured value (unit)	**core concentric-eccentric ME**<br>number of repetitions (#)
**Validity**	**nc-inter. f:** ns diff. (97)<br>**lower grade-inter. m:** ns diff. (2)
**No data on quality criteria reported:** (114)<br>**Diagnostics literature:** reliability: high level of standardization; inter-rater ICC=0.85, r=0.76***, inter-session ICC=0.98, r=0.98***; validity: corr. with sit up r=0.67***, corr. with strength measurements r=0.38*; corr. with measurements from measurement chair r=0.71 (nv) (m), r=0.52 (nv) (f) (187)	

Supplementary Table 65. Implementation variations, objects of measurement, measured values and units, data on quality criteria, and further information on fishing kicks.

**fishing kicks**	
**Object of measurement:**<br>measured value (unit)	**core concentric-eccentric ME**<br>number of repetitions (#)
**Reliability**	*inter-session (3-10 d)*<br>**adv.-elite m/f:** ICC=0.91 (86)
**Validity**	**no data m/f:** r=-.42 – -.12 (15)
**No data on quality criteria reported:** (15)<br>**Training effects:** (86)	

Supplementary Table 66. Implementation variations, objects of measurement, measured values and units, data on quality criteria, and further information on the Sorensen test.

**leg-raise**<br>lying | weight on forearms | hanging	
**Object of measurement:**<br>measured value (unit)	**core + lower leg iso. ME**<br>time to failure (s)
**Validity**	**lower grade-elite m/f:** ns diff. (14)<br>**inter-adv. m:** r=0.45**; **f:** r=0.30 (ns) (95)<br>**adv. m and nc:** ns diff. (96)
**Diagnostics literature:** lying, number of repetitions (#): reliability: inter-rater: r>0.72 (nv); inter-session reliability r=0.71 (nv); validity: corr. with strength/SE: m (r=0.72, nv), f (r=0.64, nv) (185)	
